# Changes in the Solid-, Liquid-, and Epithelium-Associated Bacterial Communities in the Rumen of Hu Lambs in Response to Dietary Urea Supplementation

**DOI:** 10.3389/fmicb.2020.00244

**Published:** 2020-02-21

**Authors:** Zhipeng Li, Chunlong Mu, Yixuan Xu, Junshi Shen, Weiyun Zhu

**Affiliations:** ^1^Laboratory of Gastrointestinal Microbiology, Jiangsu Key Laboratory of Gastrointestinal Nutrition and Animal Health, College of Animal Science and Technology, Nanjing Agricultural University, Nanjing, China; ^2^Department of Special Animal Nutrition and Feed Science, Institute of Special Animal and Plant Sciences, Chinese Academy of Agricultural Sciences, Changchun, China; ^3^National Center for International Research on Animal Gut Nutrition, Nanjing Agricultural University, Nanjing, China

**Keywords:** urea, rumen bacerial community, different microenvironment, epithelium, fermentation parameter

## Abstract

The rumen bacteria in the solid, liquid, and epithelial fractions are distinct and play important roles in the degradation of urea nitrogen. However, the effects of urea on rumen bacteria from the three fractions remain unclear. In this study, 42 Hu lambs were fed a total mixed ration based on concentrate and roughage (55:45, dry matter basis) and randomly assigned to one of three experimental diets: a basal diet with no urea (UC, 0 g/kg), a basal diet supplemented with low urea levels (LU, 10 g/kg DM), and a basal diet supplemented with high urea levels (HU, 30 g/kg DM). After an 11-week feeding trial, six animals from each treatment were harvested. Rumen metabolites levels were measured, and bacteria of the rumen solid, liquid, and epithelial fractions were examined based on 16S rRNA gene sequencing. Under urea supplementation, the concentrations of ammonia and butyrate in the rumen increased, whereas the concentration of propionate decreased. The population of total protozoa was the highest in the LU treatment. *Prevotella* 1 was the most abundant genus in all samples. The unclassified *Muribaculaceae*, bacteria within the families *Lachnospiraceae* and *Ruminococcaceae*, and *Christensenellaceae* R7 were abundant in the solid and liquid fractions. *Butyrivibrio* 2 and *Treponema* 2 were the abundant bacteria in the epithelial fraction. Principal coordinate analysis showed separation of the solid, liquid and epithelial bacteria regardless of diet, suggesting that rumen fraction had stronger influences on the bacterial community than did urea supplementation. However, the influences on the bacterial community differed among the three fractions. In the solid and liquid fractions, *Succinivibrionaceae* UCG 001 and *Prevotella* 1 showed decreased abundance with dietary urea supplementation, whereas the abundance of *Oscillospira* spp. was increased. *Howardella* spp. and *Desulfobulbus* spp. were higher in the epithelial fraction of the UC and LU treatments relative to HU treatment. Comparisons of predictive function in the rumen solid, liquid, and epithelial fractions among the three treatments also revealed differences. Collectively, these results reveal the change of the rumen bacterial community to dietary urea supplementation.

## Introduction

The ruminant livestock is an important contributor to the agricultural sector due to its production of meat and milk for human consumption; however, it is estimated that global meat and milk production will have to increase by more than 60% to meet the needs of the growing population (Huws et al., 2018). Moreover, ruminant livestock are a source of environmental pollution, excreting approximately 70% of ingested nitrogen (Huws et al., 2018). Therefore, the improvement of ruminant feed utilization has both economic and environmental benefits. For ruminants, the type and quality of protein feed play important roles in animal production because they affect the productivity of meat and milk (Schwab and Broderick, 2017). In addition, the availability of high-quality protein feed is challenged by land constraints. Thus, efforts aimed at increasing protein utilization efficiency will have considerable influences on ruminant livestock production.

In the rumen, microbiota degrade the feed protein into ammonia, which is used to synthesize the microbial proteins required for animal growth; they contribute up to 55% of the protein absorbed in the duodenum in lactating cattle (Schwab and Broderick, 2017; Huws et al., 2018). Therefore, the rumen microbiota are a key factor affecting protein utilization efficiency. The released ammonia in the rumen can be absorbed across the epithelium into the liver and then detoxified to urea, which is then recycled into the rumen and rapidly hydrolyzed to ammonia by ureases from ureolytic bacteria (Patra, 2015; Jin et al., 2017). Therefore, urea is not only a cost effective non-protein nitrogen (NPN) source that provides ammonia, which is obligately required by the fiber-digesting bacteria, but also acts a chemical component that can be measured to study the mechanisms underlying NPN metabolism by the rumen microbiota.

Recent studies indicate that the rumen microbial ecosystem is composed of three communities associated with different microenvironments: a solid-, a liquid-, and an epithelium-associated bacterial community (Cho et al., 2006; Sadet et al., 2007; Liu et al., 2016; De Mulder et al., 2017; Schären et al., 2017). The solid-adherent bacteria play key roles in fiber digestion (McAllister et al., 1994). The liquid-associated bacteria transmit bacteria from the solid-adherent biofilms to newly ingested feed (De Mulder et al., 2017). The epithelial community is diverse and distinct from the solid- and liquid-associated bacterial communities; it is associated with volatile fatty acid (VFA) fermentation, oxygen consumption, urea hydrolysis, and recycling of nitrogen and tissue (Cheng et al., 1979; Wallace et al., 1979). Although previous studies have revealed that dietary urea affects the rumen bacteria and methanogens of finishing bulls (Zhou et al., 2017) and metabolism in the rumen of dairy cows (Jin et al., 2018), it is unclear how urea supplementation affects the solid-, liquid-, and epithelium-associated bacterial communities. Additionally, a recent study suggested that rumen bacteria are specialized on an ecological basis with respect to nutrient utilization (Shaani et al., 2018). In addition, it has been documented that the ureolytic bacterial communities in the solid and liquid fractions of the rumen are different from the ureolytic bacterial community in the epithelial fraction (Jin et al., 2017). Furthermore, the rumen epithelial bacteria were found to remain largely unchanged in community structure when the feed was transitioned from a silage- and concentrate-based ration (total mixed ration, TMR) to pasture (Schären et al., 2017). Therefore, we hypothesize that the structure of the bacterial community in the solid, liquid and epithelial fractions in the rumen may be differently altered upon dietary supplementation with urea.

Therefore, the present study aimed to (1) examine the changes in the main fermentation parameters in rumen contents induced by exogenous urea supplementation in Hu lambs and (2) reveal the effects of urea supplementation on the bacterial communities and the predictive functions of the solid, liquid, and epithelial fractions by performing high-throughput sequencing of the 16S rRNA gene.

## Materials and Methods

### Experimental Design, Animals and Diets

The experiment was conducted at a Hu sheep breeding farm in Jiangsu Province, China, with a randomized complete block design. A total of 42 male Hu lambs were assigned to three blocks according to initial body weight (24.3 ± 1.7 kg). The Hu lambs in each block were fed a TMR based on concentrate and roughage [55:45, dry matter (DM) basis] and randomly assigned to one of three experimental diets (Table 1): a basal diet with no urea (UC, 0 g/kg DM), a basal diet supplemented with a low concentration of urea (LU, 10 g/kg DM), and a basal diet supplemented with a high concentration of urea (HU, 30 g/kg DM). Each dietary treatment included fourteen Hu lambs. All diets met the energy requirements for meat-producing sheep weighing 25 kg, with an assumed average daily gain (ADG) of 200 g (Ministry of Agriculture and Rural Affairs of the People’s Republic of China, 2004). The crude protein (CP) content in the diets of the UC and LU treatment groups was less than the requirement for meat-producing sheep, whereas that in the diet for the HU treatment group was more than the required amount. In our previous study, quadratic effects of urea supplementation on DM intake (DMI) and ADG were observed, and the LU treatment (corresponding to the typical inclusion level) yielded the highest DMI and ADG among the treatments (Xu et al., 2019).

**TABLE 1:** Ingredients and chemical compositions of the experimental diets.

Item	UC	LU	HU
**Ingredient, (g/kg) DM**			
Corn silage	250.0	247.5	242.7
Peanut vine	200.0	198.0	194.2
Corn grain	420.0	415.8	407.8
Soybean meal	40.0	39.6	38.8
Wheat bran	40.0	39.6	38.8
Premix^1^	50.0	49.5	48.5
Urea	0.0	10.0	30.0
**Nutrient composition**			
Crude protein (g/kg)	115.9	144.9	200.6
Neutral detergent fiber (g/kg)	326.7	331.2	328.3
Acid detergent fiber (g/kg)	203.9	213.3	208.5
Ether extract (g/kg)	30.8	31.2	31.7
Ash (g/kg)	91.2	91.2	90.9

*^1^Formulated to provide (per kilogram of DM): vitamin A, 1,320,000 IU; vitamin D3, 264,000 IU; vitamin E, 7,200 IU; Cu, 4,800 mg; Co, 73 mg; I, 144 mg; Mn, 6,480 mg; Zn, 9,600 mg; Se, 84 mg; Fe, 6,480 mg; and Mg, 7,920 mg.*

Every two lamb were reared in an individual, indoor pen (4 × 4 m) with wooden slatted floors, were offered a TMR twice daily (at 07:00 h and 19:00 h) and had free access to drinking water. The experiment was conducted over 11 weeks, with 1 week of adaptation followed by 10 weeks of dietary treatment. The experimental procedures and approaches in this study were approved by the Animal Care and Use Committee of Nanjing Agricultural University.

### Sample Collection

At the end of the experiment, the final body weights of 6 Hu lambs from each treatment were recorded, and then, the sheep were slaughtered according to the procedures of the Animal Care and Use Committee of Nanjing Agricultural University (Protocol number: SYXK2017-0007).

The rumen content of each Hu lamb was first homogenized by hand using disposable polyethylene gloves and then mixed to reduce localized effects. To obtain the liquid and solid samples, the whole rumen contents were strained through four layers of cheesecloth. Approximately 30 ml of strained liquid and the remaining pellets, representing the solid fraction, were collected in sterilized tubes. The pH of the rumen fluid was immediately measured using a portable pH meter (Ecoscan pH 5, Eutech Instruments, Singapore). To obtain the epithelial samples, the rumen walls were rinsed with cold sterile saline solution (0.9% w/v NaCl) three times after removal of the rumen contents. Epithelial samples from an approximately 1 × 1 cm area of the rumen epithelium were obtained via scraping with a sterilized glass slide. The rumen solid, liquid, and epithelial samples were immediately frozen in liquid nitrogen and then stored at –80°C until further analysis.

### Measurement of Rumen Fermentation Parameters

To measure the rumen fermentation parameters, 0.2 ml of 25% HPO_3_ was added to 1 ml of rumen fluid, and the VFA levels were then measured using gas chromatography (7890A, Agilent, United Kingdom) as previously described by Mao et al. (2008). Another 1 ml of rumen fluid was used to determine the concentration of NH_3_-N (ammonia) using a colorimetric method (Chaney and Marbach, 1962).

### DNA Extraction, PCR Amplification, Library Construction and Sequencing

Microbial genomic DNA was extracted from the rumen solid, liquid, and epithelial samples according to a bead-beating method (Yu and Morrison, 2004) using a mini-bead beater (Biospec Products, Bartlesville, OK, United States). The DNA integrity and quantity were determined using 1.0% agarose gel electrophoresis and a NanoDrop ND-1000 instrument (Thermo Scientific, Wilmington, DE, United States).

To identify the rumen bacteria in the three fractions, the primers 341F (5′-CCTACGGGAGGCAGCAG-3′) and 806R (5′-GGACTACHVGGGTWTCTAAT-3′) were used to amplify the bacterial 16S rRNA gene V4 region (Human Microbiome Project Consortium, 2012). PCR was conducted in triplicate, and the products were purified using the QIAquick PCR Purification Kit (Qiagen, CA, United States). The purified amplicons were quantified using a QuantiFluor^®^ -P fluorometer (Promega, CA, United States) and then pooled into one sample based on equimolar concentrations. Finally, the obtained amplicons were sequenced on an Illumina MiSeq platform to produce 250-bp paired-end reads.

### Sequences Analysis

The paired-end sequences were first assembled into contiguous sequences (contigs) using FLASH (Magoč and Salzberg, 2011) and then used for standard quality control by applying the default parameters in QIIME 1.9.1 (Caporaso et al., 2010). Then, the retained sequences were clustered into operational taxonomic units (OTUs) using UPARSE at 97% sequence identity (Edgar, 2013). Potential chimeras were identified and removed using UCHIME (Edgar et al., 2011). The most abundant sequences within each OTU were selected as the representative sequences and applied for the taxonomic classification based on the SILVA database (version 123) (Quast et al., 2013) using the RDP classifier with a 0.80 confidence threshold (Wang et al., 2007). The representative sequences within each OTU were aligned using MUSCLE (Edgar, 2004), and the alignment was used to construct a phylogenetic tree using FastTree (Price et al., 2009). Singletons were removed, and the sequences from each sample were then subsampled to the minimum numbers to decrease the effects of sequencing depth. The Shannon and Chao1 indices were calculated using QIIME 1.9.1 (Caporaso et al., 2010). Finally, we used phylogenetic investigation of communities by reconstruction of unobserved states (PICRUSt) to predict functional profiles of rumen microbiota resulting from reference-based OTU picking against the Greengenes database (Langille et al., 2013). The predicted genes were then summarized according to Kyoto Encyclopedia of Genes and Genomes (KEGG) pathways.

Principal coordinates analysis (PCoA) was performed and group differences based on unweighted UniFrac distance, weighted UniFrac distance and Bray–Curtis dissimilarity matrix were determined to reveal the differences in the bacterial communities across the three treatments. Analysis of similarities (ANOSIM) was performed to indicate group similarity, where 0 = indistinguishable and 1 = dissimilar (Fierer et al., 2010). Adonis was employed to describe the strengths and significance of the differences among the microbial communities. For ANOSIM and Adonis analyses, the *p*-values were determined based on 999 permutations. The sequences from the present study have been deposited in the SRA database under accession number PRJNA541835.

### Quantitative Real-Time PCR

The quantitative PCR was performed on a ABI 7300 real-time PCR System (Life Technologies, CA, United States) using SYBR Premix Ex Taq dye (TaKaRa Biotechnology, Dalian, China). The protozoal 18S rRNA primer (Sylvester et al., 2004) reported in previous study was used for the quantitative PCR. Each 20 μl reaction mixture contained 10 μl SYBR Premix Ex TaqTM (TaKaRa Biotechnology, Dalian, China), 0.4 μl of each primer (10 μM), 0.4 μl ROX Reference Dye (TaKaRa Biotechnology, Dalian, China), 6.8 μl of nuclease-free water and 2 μl of the template. Copies of 18S rRNA gene was quantified in triplicate. A standard curve was prepared by using a 10-fold serial dilutions of purified plasmid DNA containing the 18S rRNA gene sequence. The total numbers of gene copies were expressed as log_10_ numbers of marker loci gene copies per gram of sample.

### Statistical Analysis

Statistic analyses of the rumen fermentation parameters were performed using the PROC MIXED procedure of SAS 9.4 (SAS Institute Inc., Cary, NC, United States), and differences were considered to be statistically significant when the *p*-values were ≤0.05. For the comparison of bacterial genera and alpha diversity indices among the three rumen fractions under the three treatments, we used the Aligned Ranking Transform in R software and then used the Wilcoxon rank-sum test to analyze the difference between groups when the interaction was significant. All *p* values were corrected using the Benjamini–Hochberg false discovery rate (*q*-value < 0.05), and *p* < 0.05 was considered statistically significant. Values are expressed as the mean ± standard deviation (SD) unless otherwise indicated.

## Results

### Rumen Fermentation Parameters of the Lambs in the Three Treatment Groups

Total VFA concentration and pH did not significantly differ among the three treatments (Table 2). The concentrations of ammonia (*p* < 0.01) and butyrate (*p* = 0.04) were increased with urea supplementation relative to the concentration under UC treatment, whereas the molar concentration of propionate was decreased (*p* = 0.04). In addition, the molar concentration of isovalerate in the UC and LU treatments was significantly lower than that in the HU treatment (*p* < 0.01).

**TABLE 2:** Differences in the rumen fermentation parameters of lambs among the three treatments.

Item	UC	LU	HU	SEM^1^	*p*-value
Ruminal pH	5.35	5.52	5.67	0.07	0.17
Ammonia (mg/dL)	5.86^b^	10.76^b^	25.99^a^	2.90	<0.01
Acetate (mM)	71.8	70.8	73.6	2.05	0.87
Propionate (mM)	37.3^a^	32.0^ab^	22.6^b^	2.51	0.04
Butyrate (mM)	9.86^b^	11.62^b^	14.65^a^	0.86	0.04
Valerate (mM)	1.09	1.19	1.29	0.06	0.36
Isobutyrate (mM)	0.73	0.74	1.07	0.06	0.06
Isovalerate (mM)	1.17^b^	1.16^b^	1.91^a^	0.12	<0.01
Total VFAs (mM)	122.0	117.4	115.0	3.62	0.75

*^1^Standard error of means. ^*a,b*^Values within the same row with different superscripts are significantly different (*p* < 0.05).*

### Summary of Sequence Analysis of the Bacterial 16S rRNA Gene

A total of 842,698, 744,994, and 777,235 high-quality 16S rRNA gene sequences were obtained from the solid, liquid, and epithelial samples, respectively. On average, 46,816, 41,388, and 43,179 sequences were generated for each sample from the solid, liquid and epithelial samples, respectively. After subsampling, based on 97% sequence similarity, a total of 3,636, 3,816, and 2,971 OTUs were obtained for the solid (mean = 1,748), liquid (mean = 1,734) and epithelial (mean = 1,486) samples, respectively.

The results showed that the sequencing depth covered more than 98% of the bacterial community, ranging from 97.6% to 98.9%. The number of OTUs and Chao 1 index value were significantly higher in the solid and liquid fractions than those in the epithelial faction (Table 3). In addition, the number of OTUs and the Shannon and Chao1 index values were higher in the HU treatment than in the UC or LU treatment for all three fractions (Table 3). Moreover, within the epithelial fraction, the number of OTUs and the Chao1 index value in the HU treatment were significantly higher than those in the LU treatment.

**TABLE 3:** Comparison of diversity and richness indices among the solid, liquid, and epithelial fractions under the tree treatments.

Item	OTU numbers			Shannon			Chao 1		
			
	Solid	Liquid	Epithelium	Solid	Liquid	Epithelium	Solid	Liquid	Epithelium
UC	2084^c^	2015^c^	1609^b^	5.80	5.60	5.52	3015^c^	2897^c^	2241^b^
LU	1991^c^	1936^c^	1621^b^	5.59	5.54	5.55	2987^c^	2882^c^	2281^b^
HU	2211^c^	2243^c^	1971^a^	6.31	6.24	6.10	3101^c^	3159^c^	2655^a^
SEM^1^	316			0.08			441		
F	<0.001			0.406			<0.001		
T	0.002			<0.001			0.024		
F × T^2^	0.877			0.986			0.837		

*^1^Standard error of means. ^2^Probability of a significant effect due to rumen fractions (F), treatment (T), and their interaction (F × T). ^*a,b,c*^Values within the same row or column with different superscripts are significantly different (*p* < 0.05). UC, basal diet with no urea; LU, basal diet supplemented with a low concentration of urea (10 g/kg DM); HU, basal diet supplemented with a high concentration of urea (30 g/kg DM).*

**FIGURE 1:**
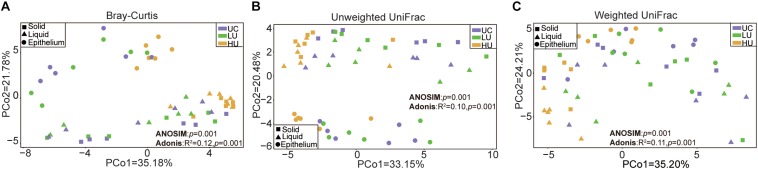
Principal coordinates analysis (PCoA) revealing the separation of the rumen bacteria in the three fractions from the three treatments based on Bray–Curtis dissimilarity matrix **(A)**, unweighted UniFrac distance **(B)**, and weighted UniFrac distance **(C)**. UC, basal diet with no urea; LU, basal diet supplemented with a low concentration of urea (10 g/kg DM); HU, basal diet supplemented with a high concentration of urea (30 g/kg DM).

The PCoA results showed that the bacterial communities from the three fractions were separated from one another based on Bray–Curtis dissimilarity matrix (Figure 1A, ANOSIM: *p* = 0.001; Adonis: *p* = 0.001), unweighted UniFrac distance (Figure 1B, ANOSIM: *p* = 0.001; Adonis: *p* = 0.001), and weighted UniFrac distance (Figure 1C, ANOSIM: *p* = 0.001; Adonis: *p* = 0.001).

In order to reveal the difference among the three fractions, we compared the relative abundance of bacterial genus (Table 4). The prevalence of *Prevotella* 1, the unclassified bacteria within the family *Muribaculaceae*, *Christensenellaceae* R7, *Ruminococcaceae* NK4A214, *Lachnospiraceae* NK3A20, the unclassified bacteria within the family *Lachnospiraceae*, *Ruminococcaceae* UCG 014, and *Ruminococcus* 2 were higher in the rumen solid and liquid fractions than in the epithelium fraction. Whereas, the genera *Treponema* 2, *Butyrivibrio* 2, *Desulfobulbus*, and *Campylobacter* were higher in the epithelium fraction than in the solid and liquid fractions.

**TABLE 4:** The effect of rumen fractions on the relative abundances (%) of bacterial genus.

Genus	Solid	Liquid	Epithelium	SEM^1^	*p-*value^2^		
					
					*F*	*T*	F × T
Prevotella 1	18.84^ab^	19.39^a^	14.11^c^	1.03	0.032	<0.001	0.053
Muribaculaceae unclassified	5.60^ab^	6.32^a^	3.34^c^	0.47	0.003	0.640	0.154
Christensenellaceae R7	5.02^a^	4.15^ab^	2.75^b^	0.29	<0.001	0.004	0.140
Treponema 2	3.94^b^	3.33^b^	5.82^a^	0.42	0.022	0.009	0.911
Succiniclasticum	3.72^a^	1.74^b^	1.62^b^	0.22	<0.001	0.009	0.067
Ruminococcaceae NK4A214	2.97^a^	3.30^ab^	1.46^c^	0.19	<0.001	0.012	0.035
Ruminococcus 1	2.12^a^	1.15^b^	0.46^c^	0.11	<0.001	0.500	0.616
Prevotellaceae UCG 001	2.00^b^	2.41^b^	7.26^a^	0.44	<0.001	0.008	0.020
Lachnospiraceae NK3A20	1.55^a^	1.13^b^	0.90^b^	0.06	<0.001	0.794	0.820
Lachnospiraceae unclassified	1.49^ab^	2.13^a^	0.56^c^	0.31	<0.001	<0.001	0.007
Prevotellaceae UCG 003	1.46^ab^	1.78^a^	1.14^b^	0.10	0.011	0.716	0.001
Ruminococcaceae UCG 014	1.34^ab^	1.83^a^	0.38^c^	0.13	<0.001	0.031	0.174
Saccharofermentans	1.31^a^	0.62^b^	0.23^c^	0.08	<0.001	0.002	0.056
Prevotellaceae NK3B31	1.27^a^	0.45^bc^	0.50^b^	0.07	<0.001	0.002	0.083
Ruminococcus 2	1.25^ab^	1.95^a^	0.45^c^	0.18	<0.001	0.009	0.444
Eubacterium ruminantium	1.09^a^	0.60^b^	0.16^c^	0.06	<0.001	0.901	0.911
Butyrivibrio 2	0.81^b^	0.58^c^	7.19^a^	0.48	<0.001	0.426	0.123
Lachnospiraceae AC2044	0.77^a^	0.50^b^	0.19^c^	0.05	<0.001	<0.001	0.074
Lachnospiraceae NK4A136	0.75^a^	0.36^b^	0.21^c^	0.04	<0.001	0.045	0.103
Bacteroidales RF16 unclassified	0.63^c^	4.47^a^	1.55^b^	0.37	<0.001	0.913	0.790
Acetitomaculum	0.63^a^	0.42^b^	0.34^bc^	0.03	<0.001	0.771	0.521
Prevotellaceae UCG 004	0.58^a^	0.26^c^	0.38^b^	0.03	<0.001	0.700	0.014
Veillonellaceae UCG 001	0.37^b^	0.65^a^	0.23^bc^	0.05	<0.001	0.038	0.374
Eubacterium coprostanoligenes	0.35^b^	0.78^a^	0.18^c^	0.06	<0.001	<0.001	<0.001
Selenomonas 1	0.21^b^	0.63^a^	0.16^bc^	0.05	<0.001	0.001	0.227
Erysipelotrichaceae UCG 004	0.11^c^	0.51^a^	0.19^b^	0.04	<0.001	0.054	0.329
Ruminococcaceae UCG 001	0.11^b^	0.51^a^	0.04^c^	0.06	<0.001	0.007	0.168
Bacteroidales BS11	0.32^ab^	0.27^bc^	0.71^a^	0.07	0.031	0.943	0.060
Anaerovorax	0.31^ab^	0.18^c^	0.57^a^	0.05	<0.001	0.043	0.727
Family XIII AD3011	0.28^b^	0.19^c^	0.50^a^	0.03	<0.001	0.578	0.084
Lachnospiraceae UCG 008	0.18^b^	0.12^bc^	0.76^a^	0.06	<0.001	0.107	0.089
Prevotellaceae unclassified	0.09^c^	0.15^b^	0.65^a^	0.09	<0.001	0.073	0.450
Alloprevotella	0.09^c^	0.40^ab^	0.52^a^	0.04	<0.001	0.909	0.428
Fretibacterium	0.01^bc^	0.03^b^	0.51^a^	0.04	<0.001	0.510	0.653
Desulfobulbus	0.00^c^	0.03^b^	2.83^a^	0.22	<0.001	<0.001	<0.001
Campylobacter	0.00^bc^	0.00^b^	0.55^a^	0.05	<0.001	<0.001	0.003
Bacteroidales unclassified	0.47^b^	0.30^c^	0.76^a^	0.04	<0.001	0.348	0.938

*^1^Standard error of means. ^2^Probability of a significant effect due to rumen fractions (F), treatment (T), and their interaction (F × T). ^*a,b,c*^Values within the same row with different superscripts are significantly different (*p* < 0.05).*

To assess functional profiles of rumen microbiota, we applied PICRUSt to predict the potential functions and compared the difference among the three fractions (Table 5). At KEGG level 2, the relative abundance of amino acid metabolism, carbohydrate metabolism, replication and repair, and translation pathways were significantly higher in the rumen solid and liquid fractions than in the rumen epithelial fraction. However, the pathways of energy metabolism, cell motility, and signal transduction accounted for higher abundance in rumen epithelial fraction than in the rumen solid and liquid fractions. In addition, because rumen microbiota are clearly separated among the three fractions, thus we compared the bacterial community composition and potential function in the rumen solid, liquid, and epithelial fractions, respectively.

**TABLE 5:** The effect of fractions on the predictive function (%) of rumen microbiota.

Level 2	Solid	Liquid	Epithelium	SEM^1^	*p-*value^2^		
					
					*F*	*T*	F × T
Amino acid metabolism	10.17^ab^	10.25^a^	10.07^b^	0.03	0.023	0.754	0.092
Biosynthesis of other secondary metabolites	0.99^ab^	1.01^a^	0.97^b^	0.01	0.016	0.851	0.147
Carbohydrate metabolism	10.14^ab^	10.31^a^	9.88^c^	0.04	<0.001	0.001	0.937
Cell motility	2.14^b^	2.00^bc^	2.61^a^	0.06	<0.001	0.046	0.011
Cellular processes and signaling	3.87^ab^	3.87^a^	3.74^b^	0.02	0.004	0.041	0.651
Digestive system	0.06^b^	0.06^a^	0.05^bc^	0.00	0.042	0.346	0.055
Endocrine system	0.33^ab^	0.34^a^	0.32^c^	0.00	0.030	0.028	0.685
Energy metabolism	6.07^b^	6.05^bc^	6.24^a^	0.03	0.002	0.007	0.952
Environmental adaptation	0.15^b^	0.15^bc^	0.16^a^	0.00	0.026	0.007	0.027
Enzyme families	2.19^ab^	2.19^a^	2.17^c^	0.01	0.025	0.098	0.358
Genetic information processing	2.73^b^	2.72^bc^	2.78^a^	0.01	<0.001	0.010	0.193
Glycan biosynthesis and metabolism	2.61	2.75	2.69	0.03	0.020	0.001	0.011
Immune system diseases	0.04^bc^	0.04^b^	0.05^a^	0.00	<0.001	0.002	0.053
Metabolic diseases	0.11^b^	0.12^a^	0.10^c^	0.00	<0.001	0.124	0.754
Nucleotide metabolism	4.33^b^	4.38^a^	4.28^bc^	0.01	0.007	0.315	0.045
Replication and repair	9.65^ab^	9.73^a^	9.38^c^	0.04	<0.001	0.066	0.105
Signal transduction	1.44^b^	1.39^c^	1.62^a^	0.02	<0.001	0.175	0.001
Transcription	2.60^a^	2.54^ab^	2.42^c^	0.02	<0.001	<0.001	0.019
Translation	6.35^ab^	6.39^a^	6.26^c^	0.02	0.017	0.115	0.190
Xenobiotics biodegradation and metabolism	1.53^b^	1.52^bc^	1.64^a^	0.01	<0.001	0.076	0.951

*^1^Standard error of means. ^2^Probability of a significant effect due to rumen fractions (F), treatment (T), and their interaction (F × T). ^*a,b,c*^Values within the same row with different superscripts are significantly different (*p* < 0.05).*

### Bacterial Community and Potential Function in the Solid Fraction Under the Three Treatments

**FIGURE 2:**
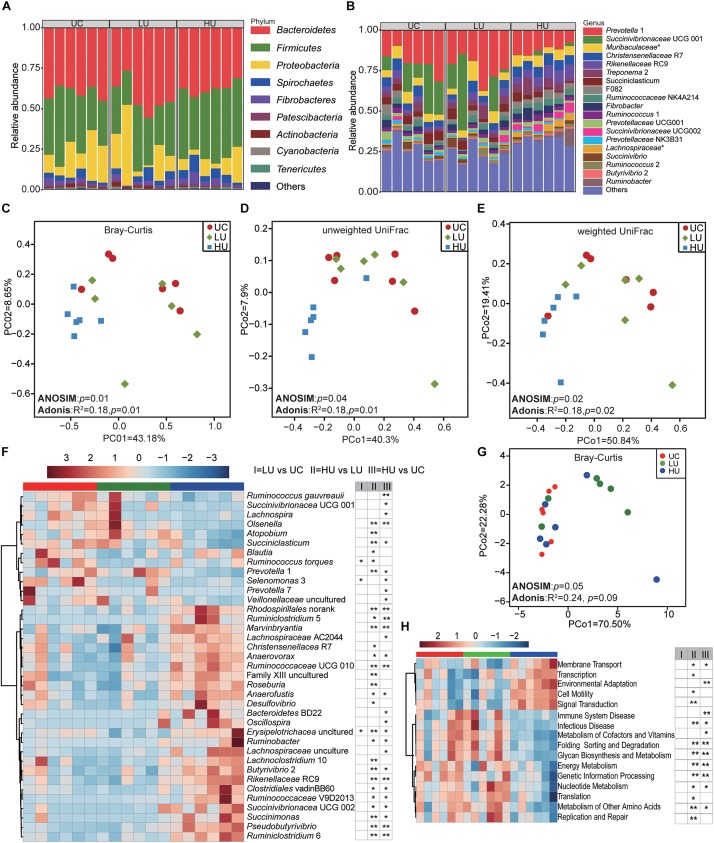
Bacterial community compositions and potential function in the rumen solid fractions under the three treatments. Bacterial compositions in the rumen solid fractions of the UC, LU, and HU treatments at the phylum **(A)** and genus **(B)** levels. PCoA revealing the separation of the microbial communities in the rumen solid fractions among the three treatments based on Bray–Curtis dissimilarity matrix **(C)**, unweighted UniFrac distance **(D)** and weighted UniFrac distance **(E)**. Heatmap **(F)** showing significant differences of bacterial genera in the solid fractions among the UC, LU, and HU treatments. PCoA **(G)** plot revealing differences in predicted microbial functions based on Bray–Curtis dissimilarity matrix. Heatmap **(H)** revealing the differences of the predictive function profiles at KEGG level 2 in the rumen solid fractions among the three treatments. * *p* < 0.05, ** *p* < 0.01. UC, basal diet with no urea; LU, basal diet supplemented with a low concentration of urea (10 g/kg DM); HU, basal diet supplemented with a high concentration of urea (30 g/kg DM).

A total of 18, 16, and 17 phyla were identified in the rumen solid fraction in the UC, LU, and HU treatment groups, respectively (Figure 2A). The phylum *Bacteroidetes* predominated the rumen solid fraction of the UC (40.0 ± 4.0%) and LU (43.2 ± 10.2%) treatments, followed by the phylum *Firmicutes* (UC = 34.7 ± 8.4%, LU = 29.8 ± 6.9%). However, in the HU treatment, *Firmicutes* (41.5 ± 4.7%) was the most abundant phylum in the solid fraction, followed by the phylum *Bacteroidetes* (37.7 ± 3.9%). *Proteobacteria* was the third most abundant phylum in the rumen solid fraction regardless of diet (UC = 16.9 ± 9.9%, LU = 17.8 ± 10.9%, HU = 9.3 ± 6.9%).

A total of 190, 195, and 211 bacterial genera were identified in the UC, LU, and HU treatments, respectively (Figure 2B). In the UC and LU treatments, *Prevotella* 1 (UC = 19.9 ± 7.2%, LU = 24.1 ± 8.0%) was the most abundant genus in the rumen solid fraction, followed by *Succinivibrionaceae* UCG 001 (UC = 14.5 ± 10.6%, LU = 14.4 ± 9.1%) and the unclassified bacterium within the family *Muribaculaceae* (UC = 5.4 ± 1.6%, LU = 5.8 ± 3.3%); together, these taxa accounted for approximately 40% of the overall bacterial composition. In the HU treatment, the genus *Prevotella* 1 (12.4 ± 3.0%) was predominant in the rumen solid fraction, followed by *Rikenellaceae* RC9 (6.8 ± 1.8%), *Christensenellaceae* R7 (6.2 ± 0.6%), the unclassified bacterium within the family *Muribaculaceae* (5.5 ± 1.7%), and *Treponema* 2 (5.2 ± 2.9%); together, these taxa accounted for more than 36% of the overall bacterial composition.

The PCoA plots showed that the composition of bacterial community differed significantly among the three treatments based on Bray–Curtis dissimilarity matrix (Figure 2C, ANOSIM: *p* = 0.01; Adonis: *p* = 0.01), unweighted UniFrac distance (Figure 2D, ANOSIM: *p* = 0.04; Adonis: *p* = 0.01) and weighted UniFrac distance (Figure 2E, ANOSIM: *p* = 0.02; Adonis: *p* = 0.02). Moreover, comparison of group distances across the three treatments showed that the bacterial community differed significantly between the LU and HU treatments (Supplementary Figure S1).

We then applied the non-parametric Wilcoxon rank-sum test to conduct pair-wise comparisons among the three treatments. The result showed that a total of 37 bacterial genera were significantly different between one or more pairs of treatments (Figure 2F and Supplementary Table S1). The relative abundances of *Ruminococcus gauvreauii*, *Ruminococcus gauvreauii*, *Succinivibrionaceae* UCG 001, and *Selenomonas* 3 were significantly higher in the UC treatment than in the LU or HU treatment. The relative abundances of the genera *Prevotella* 1, *Atopobium*, and *Olsenella* were significantly higher in the LU treatment than in the UC or HU treatment, whereas the relative abundance of *Christensenellaceae* R7 was lower in the LU treatment than in the other treatments. The relative abundances of *Rikenellaceae* RC9, *Ruminobacter* spp., *Succinivibrionaceae* UCG 002, *Anaerofustis* spp., *Ruminococcaceae* UCG 010, *Succinimonas* spp., *Butyrivibrio* 2, *Pseudobutyrivibrio* spp., *Ruminococcaceae* V9D2013, *Roseburia* spp., *Desulfovibrio* spp., *Ruminiclostridium* 6, *Marvinbryantia* spp., *Anaerovorax* spp., *Ruminiclostridium* 5, and *Lachnospiraceae* AC2044 were significantly increased in the HU treatment relative to the corresponding abundances in the UC or LU treatment. However, the relative abundances of *Succiniclasticum* spp., *Lachnospira* spp., *Prevotella* 7, and the unclassified bacteria within the family *Veillonellaceae* were significantly lower in the HU treatment than in the UC or LU treatments.

The PCoA result of all KOs based on Bray-Curtis dissimilarity matrix showed that the functional profiles in the rumen solid fraction of the HU treatment tended to separate the UC and LU treatments (Figure 2G, ANOSIM: *p* = 0.05; Adonis: *p* = 0.09). Comparison of KEGG pathways at level 2 among the three treatments indicated that energy metabolism, genetic information processing and metabolism of cofactors and vitamins pathways were higher in the UC and HU treatments than those in the LU treatment, while replication and repair and translation pathways were higher in the LU treatment (Figure 2H). At KEGG level 3, a total of 81 pathways were significantly different (Supplementary Table S2). For example, the pathways of alanine, aspartate and glutamate metabolism, amino acid related enzymes, and purine metabolism were higher in the LU treatment than those in the UC and HU treatments. On the contrary, transporters pathway was lower in the HU treatment relative to that in the UC or LU treatment.

### Bacterial Community and Potential Function in the Liquid Fraction Under the Three Treatments

**FIGURE 3:**
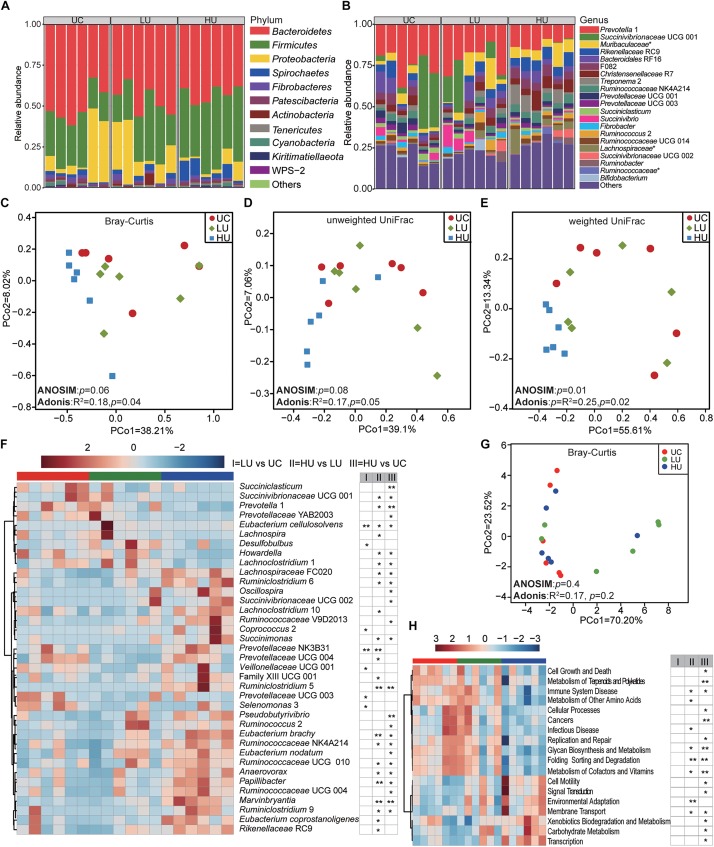
Bacterial community compositions and potential function in the rumen liquid fractions under the three treatments. Box plots showing the bacterial community compositions in the rumen liquid fractions of the UC, LU, and HU treatments at the phylum **(A)** and genus **(B)** levels. PCoA based on Bray–Curtis dissimilarity matrix **(C)**, unweighted UniFrac distance **(D)**, and weighted UniFrac distance **(E)**. Heatmap **(F)** showing differential taxa in the liquid fractions among the three treatments. PCoA **(G)** plot revealing differences in predicted microbial functions based on Bray–Curtis dissimilarity matrix. Heatmap **(H)** revealing the differences of the predictive function profiles at KEGG level 2 in the rumen solid fractions among the three treatments. * *p* < 0.05, ** *p* < 0.01. UC, basal diet with no urea; LU, basal diet supplemented with a low concentration of urea (10 g/kg DM); HU, basal diet supplemented with a high concentration of urea (30 g/kg DM).

A total of 16, 17, and 18 phyla were identified in the rumen liquid fractions from the UC, LU, and HU treatments, respectively (Figure 3A). *Bacteroidetes* (UC = 49.9 ± 10.8%, LU = 46.3 ± 9.0%, HU = 42.6 ± 5.0%) was the most abundant phylum in the three treatments, followed by the phyla *Firmicutes* (UC = 25.7 ± 6.6%, LU = 27.8 ± 10.2%, HU = 41.2 ± 7.6%) and *Proteobacteria* (UC = 16.8 ± 9.1%, LU = 17.9 ± 12.8%, HU = 4.7 ± 4.0%).

A total of 182, 183, and 183 bacterial genera were identified in the rumen liquid samples from the UC, LU, and HU treatments, respectively (Figure 3B). *Prevotella* 1 was the most abundant genus across the three treatments (UC = 24.5 ± 8.2%, LU = 21.4 ± 8.4%, HU = 12.1 ± 3.2%). In the UC and LU treatments, *Bacteroidales* RF16 (UC = 5.3 ± 4.2%, LU = 4.5 ± 3.0%) and *Succinivibrionaceae* UCG 001 (UC = 14.0 ± 9.2%, LU = 10.0 ± 8.2%) were abundant in the rumen liquid fractions. In the HU treatment, *Rikenellaceae* RC9 (6.8 ± 1.6%), *Christensenellaceae* R7 (6.3 ± 3.4%), and the unclassified bacterium within the family *Muribaculaceae* (7.4 ± 3.7%) also exhibited high prevalence.

The PCoA results showed that the bacterial community in the rumen liquid differed significantly among the three treatments based on weighted UniFrac distance (Figure 3E, ANOSIM: *p* = 0.01; Adonis: *p* = 0.02). However, the differences were not significant based on Bray-Curtis dissimilarity matrix (Figure 3C, ANOSIM: *p* = 0.06; Adonis: *p* = 0.04) or unweighted UniFrac distance (Figure 3D, ANOSIM: *p* = 0.08; Adonis: *p* = 0.05). Moreover, the group distances between LU and HU were significantly different (Supplementary Figure S2).

The relative abundances of *Succinivibrionaceae* UCG 001, *Prevotella* 1, *Succiniclasticum* spp., *Howardella* spp., *Selenomonas* 3, and *Prevotellaceae* UCG 003 were significantly higher in the UC treatment than in the LU or HU treatment (Figure 3F and Supplementary Table S3). The relative abundances of *Eubacterium cellulosolvens*, *Lachnospira* spp., *Desulfobulbus* spp., and *Lachnoclostridium* 1 in the LU treatment were significantly greater than the corresponding abundances in the UC or HU treatment. The relative abundances of *Rikenellaceae* RC9, *Ruminococcaceae* NK4A214, *Eubacterium nodatum*, *Eubacterium coprostanoligenes*, *Eubacterium brachy*, *Ruminococcaceae* UCG 010, *Prevotellaceae* NK3B31, *Lachnospiraceae* FCS020, *Marvinbryantia* spp., *Papillibacter* spp., *Succinimonas* spp., *Pseudobutyrivibrio* spp., *Ruminococcaceae* V9D2013, *Ruminiclostridium* 6, *Anaerovorax* spp., *Oscillospira* spp., *Succinivibrionaceae* UCG 002, *Lachnoclostridium* 10, *Ruminococcus* 2, *Coprococcus* 2, *Ruminiclostridium* 5, and *Ruminiclostridium* 9 were increased significantly in the HU treatment relative to those in the UC or LU treatment (Figure 3F and Supplementary Table S3).

PCoA of all KOs based on Bray-Curtis dissimilarity matrix showed that the functional profiles in the rumen liquid fraction were not significantly different among the three treatments (Figure 3G, ANOSIM: *p* = 0.4; Adonis: *p* = 0.2). However, the relative abundance of carbohydrate metabolism, and metabolism of other amino acids pathways increased with the supplementation of urea in diet, while metabolism of cofactors and vitamins pathway decreased (Figure 3H). At KEGG level 3, a total of 92 pathways were significantly different among the three treatments (Supplementary Table S4). For instance, the pathways of methane metabolism, protein digestion and absorption, and protein kinases decreased with urea supplementation in diet, while the pathways of pyruvate metabolism, valine, leucine and isoleucine degradation, and butanoate (butyrate) metabolism increased (Supplementary Table S4).

### Bacterial Community and Potential Function in the Epithelial Fraction Under the Three Treatments

**FIGURE 4:**
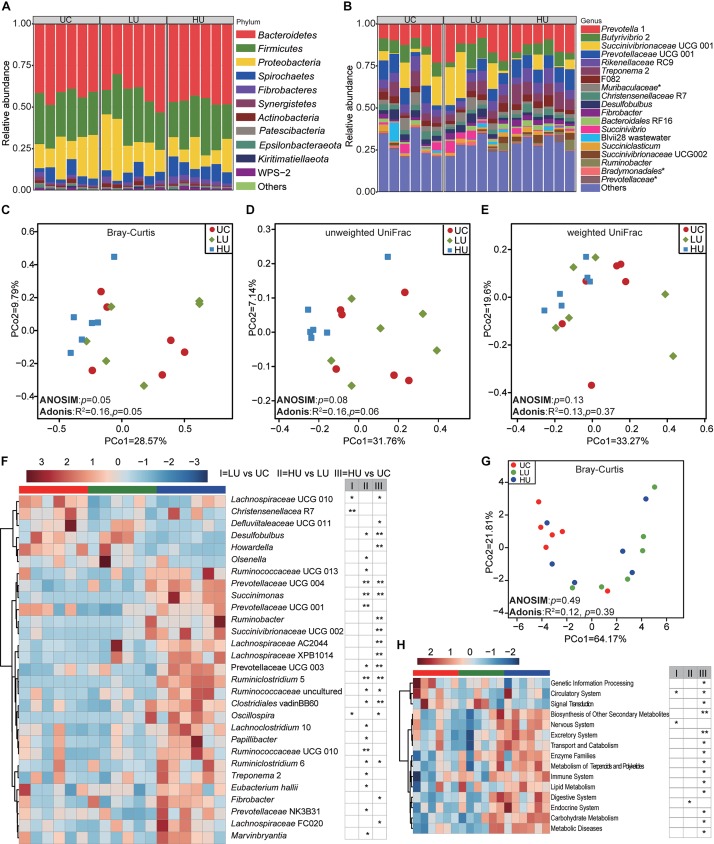
Bacterial community compositions and potential function in the rumen epithelial fractions under the three treatments. The bacterial community compositions in the rumen epithelial fractions of the UC, LU and HU treatments at the phylum **(A)** and genus **(B)** levels. Comparison of the bacterial communities of epithelial fractions based on Bray–Curtis dissimilarity matrix **(C)**, unweighted UniFrac distance **(D)** and weighted UniFrac distance **(E)**. Heatmap **(F)** showing differential taxa in the epithelium fractions among the three treatments. PCoA **(G)** plot revealing differences in predicted microbial functions based on Bray–Curtis dissimilarity matrix. Heatmap **(H)** revealing the differences of the predictive function profiles at KEGG level 2 in the rumen solid fractions among the three treatments. * *p* < 0.05, ** *p* < 0.01. UC, basal diet with no urea; LU, basal diet supplemented with a low concentration of urea (10 g/kg DM); HU, basal diet supplemented with a high concentration of urea (30 g/kg DM).

A total of 22, 19, and 21 phyla were identified in the rumen epithelial fractions from the UC, LU, and HU treatments, respectively (Figure 4A). The phylum *Bacteroidetes* (UC = 42.5 ± 3.4%, LU = 41.1 ± 7.9%, HU = 46.2 ± 2.2%) was abundant in the rumen epithelial fraction of Hu lambs, followed by the phyla *Firmicutes* (UC = 27.4 ± 3.0%, LU = 26.0 ± 7.2%, HU = 26.3 ± 5.2%) and *Proteobacteria* (UC = 18.7 ± 7.3%, LU = 22.1 ± 10.7%, HU = 12.2 ± 5.9%). In addition, the phylum *Spirochaetes* accounted for approximately 5% of the rumen epithelial fraction of each of the three treatments (UC = 5.2 ± 3.2%, LU = 5.0 ± 2.4%, HU = 8.2 ± 2.5%).

A total of 255, 244, and 256 bacterial genera were identified in the UC, LU, and HU treatments, respectively (Figure 4B). *Prevotella* 1 accounted for the highest proportion in all three treatments (UC = 12.6 ± 5.5%, LU = 15.6 ± 6.0%, HU = 13.9 ± 3.4%), followed by *Succinivibrionaceae* UCG 001 (UC = 9.7 ± 7.9%, LU = 11.2 ± 9.1%, HU = 2.7 ± 1.7%), *Butyrivibrio* 2 (UC = 7.5 ± 1.9%, LU = 7.8 ± 4.5%, HU = 6.4 ± 2.4%), *Rikenellaceae* RC9 (UC = 5.7 ± 1.6%, LU = 5.0 ± 1.6%, HU = 6.5 ± 1.9%), *Prevotellaceae* UCG 001 (UC = 8.0 ± 4.3%, LU = 4.7 ± 2.6%, HU = 9.2 ± 1.9%), and *Treponema* 2 (UC = 4.9 ± 3.1%, LU = 4.7 ± 2.2%, HU = 7.9 ± 2.5%).

The PCoA, ANOSIM and Adonis results indicated that the bacterial community in the rumen epithelium was not distinguishable among the three treatments based on Bray–Curtis dissimilarity matrix (Figure 4C, ANOSIM: *p* = 0.05; Adonis: *p* = 0.05), unweighted UniFrac distance (Figure 4D, ANOSIM: *p* = 0.08; Adonis: *p* = 0.06) and weighted UniFrac distance (Figure 4E, ANOSIM: *p* = 0.13; Adonis: *p* = 0.37); this result was supported by the comparison of group distances (Supplementary Figure S3).

The comparison of bacterial genera among the three treatments showed that the relative abundances of *Desulfobulbus* spp., *Howardella* spp., *Christensenellaceae* R7, and *Lachnospiraceae* UCG 010 were significantly higher in the UC treatment than in the LU or HU treatment (Figure 4F and Supplementary Table S5). The relative abundance of *Olsenella* spp. was significantly greater in the LU treatment than in the UC or HU treatment, whereas that of *Marvinbryantia* spp. was significantly lower in the LU treatment than in the UC or HU treatment (Figure 4F and Supplementary Table S5). The relative abundances of *Prevotellaceae* UCG 001, *Succinivibrionaceae* UCG 002, *Ruminobacter* spp., *Prevotellaceae* UCG 003, *Succinimonas* spp., *Prevotellaceae* NK3B31, *Ruminococcaceae* UCG 010, *Ruminococcaceae* UCG 013, *Prevotellaceae* UCG 004, *Lachnospiraceae* XPB1014, *Lachnospiraceae* UCG 010, *Papillibacter* spp., *Oscillospira* spp., *Treponema* 2, *Fibrobacter* spp., *Ruminiclostridium* 5, and *Ruminiclostridium* 6 were higher in the HU treatment than in the UC and LU treatments (Figure 4F and Supplementary Table S5).

PCoA result of all KOs showed that the microbial function in the rumen epithelial fraction from the HU treatment tended to differ from the UC and LU treatments, although the difference was not significant (Figure 4G, ANOSIM: *p* = 0.49; Adonis: *p* = 0.39). At KEGG level 2, the relative abundance of biosynthesis of other secondary metabolites pathway increased with the supplementation of urea in diet, while carbohydrate metabolism pathway decreased (Figure 4H). At KEGG level 3, a total of 54 pathways were significantly different in the rumen liquid fraction among the three treatments (Supplementary Table S6). The pathways of phenylalanine, tyrosine and tryptophan biosynthesis, and D-arginine and D-ornithine metabolism decreased from UC to HU treatments, while cysteine and methionine metabolism pathway increased (Supplementary Table S6).

### Quantification of Protozoal Density

No interaction (*p* = 0.82) between fractions and treatments was detected with respect to the absolute abundance of total protozoa (Table 6). The population of protozoa in the LU treatment was higher than that in the UC and HU treatments (*p* < 0.01). Moreover, the liquid fraction had a higher (*p* < 0.01) protozoal population than that in the solid and epithelial fractions regardless of diet.

**TABLE 6:** Population of total protozoa in the rumen solid, liquid, and epithelium fractions among the three treatments (log_10_ copy number of 18S RNA gene per gram of sample).

Protozoa	UC	LU	HU	SEM^1^	*p*-value^2^		
					
					*F*	*T*	F × T
Solid	6.80	7.24	6.92				
Liquid	7.42^a^	8.05^b^*	7.54^ab^	0.11	<0.001	<0.001	0.82
Epithelium	7.09	7.33	7.02				

^1^Standard error of means. ^2^Probability of a significant effect due to rumen fractions (F), treatment (T), and their interaction (F × T). ^*a,b*^Values within the same row with different superscripts are significantly different (*p* < 0.05). * Within the same column means differ significantly (*p* < 0.05). UC, basal diet with no urea; LU, basal diet supplemented with a low concentration of urea (10 g/kg DM); HU, basal diet supplemented with a high concentration of urea (30 g/kg DM).

## Discussion

### Differences in the Rumen Fermentation Parameters Among the Three Treatments

In the present study, the ammonia level in the rumen was increased with urea supplementation (Table 2), which is consistent with previous results (Spanghero et al., 2018). This result can be attributed to diverse ureolytic bacteria that do not limit the conversion of urea to ammonia (Cook, 1976; Jin et al., 2017), and the increased number of rumen protozoa in the LU treatment in comparison with UC treatment (Table 6). Rumen protozoa play an important role in the bacterial protein breakdown (Williams and Coleman, 1992), and the protozoal elimination results in a decrease in rumen ammonia based on a meta-analysis (Newbold et al., 2015). Previous study reported that the maximum concentration of microbial protein in the rumen was associated with an ammonia concentration of 8.8 mg/dL (Hume et al., 1970), which is comparable to that the concentration in the LU treatment (10.76 mg/dL). The present result is also consistent with the finding that DMI and ADG were highest in the LU treatment among different treatments (Xu et al., 2019). However, the concentration of ammonia in the rumen significantly increased from 5.86 mg/dL in the UC treatment to 25.99 mg/dL in the HU treatment (Table 2). Rumen ammonia can be absorbed via simple diffusion and via potassium channels and some transport proteins (Abdoun et al., 2006). Ruminants may display signs and symptoms of ammonia toxicity when the ammonia concentration in the rumen is above 100 mg/dL (Owens and Bergen, 1983). Moreover, the molar concentration of total VFA did not significantly differ among the three treatments, which is consistent with previous findings in beef steers administered slow-release urea (Taylor-Edwards et al., 2009). Together, these findings suggest urea supplementation affected the ammonia metabolism in rumen.

The molar concentrations of butyrate and isovalerate were higher in the HU treatment than in the UC and LU treatments (Table 2). Similarly, Meng et al. (2000) and Spanghero et al. (2018) documented that butyrate production increased during *in vitro* rumen fermentation. Moreover, Jin et al. (2018) found that valine, leucine, and isoleucine metabolism were enhanced in the rumen of dairy cattle fed urea. Previous studies have suggested that isovalerate is derived from branched-chain amino acids, such as valine and isoleucine (Allison, 1978). Interestingly, the pathways of butyrate metabolism, and valine leucine and isoleucine degradation were also higher in the rumen liquid of HU treatment than in the UC and LU treatments (Supplementary Table S4). These results suggest that the metabolism of butyrate and branched-chain amino acids is affected by urea supplementation.

### Differences in Bacterial Community Structure Among the Solid, Liquid, and Epithelial Fractions

To understand the changes in rumen metabolism, we examined the rumen microbiota in the solid, liquid and epithelial fractions. The phyla *Bacteroidetes*, *Firmicutes*, and *Proteobacteria* were abundant bacteria in the Hu lamb rumen regardless of diet or fraction (Figures 2A, 3A, 4A), which is consistent with previous findings on the global rumen microbiota (Henderson et al., 2015) and indicates the existence of a core rumen microbiota. In addition, we found that *Prevotella* was the most abundant genus in the three fractions (Figures 2B, 3B, 4B). This result is consistent with findings regarding the rumen solid and liquid fractions of dairy cattle fed ryegrass or white clover (Bowen et al., 2018) and a TMR (forage:concentrate = 70:30, forage = prewilted grass and maize silage) (De Mulder et al., 2017) and the rumen contents and epithelium of dairy cattle fed a TMR (forage:concentrate = 55:45, forage = corn silage and corn stover) (Liu et al., 2016). *Prevotella* represents one of the most abundant genera in the rumen; this genus exhibits genetic and metabolic diversity (Bekele et al., 2010) and plays roles in carbohydrate utilization (Dehority, 1966; Cotta, 1992; Gardner et al., 1995; Kabel et al., 2011), nitrogen metabolism (Kim et al., 2017), and fiber degradation (Mayorga et al., 2016). The results of these study suggest the importance of *Prevotella* spp. in the rumen microbial community. However, in contrast to our findings, Schären et al. (2017) found that the family *Lachnospiraceae* was predominant in the rumen epithelium of German Holsteins fed a TMR (35% corn silage, 35% grass silage, 30% concentrate). This discrepancy may be attributed to differences in dietary composition (Henderson et al., 2015) and sample collection approaches (Li et al., 2009).

At the genus level, the present study found that the unclassified bacteria within the families *Muribaculaceae* and *Lachnospiraceae*, *Christensenellaceae* R7, *Ruminococcaceae* NK4A214, *Lachnospiraceae* NK3A20, *Ruminococcaceae* UCG 014, and *Ruminococcus* 2 were abundant in the solid and liquid fractions (Table 4). These bacteria have also been observed in the solid and liquid fractions of dairy cattle (De Mulder et al., 2017; Schären et al., 2017) and yak (Ren et al., 2019). The bacteria within the family *Muribaculaceae* encode enzymes that degrade plant glycans (hemicellulose and pectin) and host-derived glycans; they also exhibit specificity in nitrogen utilization and harbor a specific urease (Ormerod et al., 2016; Lagkouvardos et al., 2019). Members of the *Christensenellaceae* family contain enzymes, such as α-arabinosidase, β-galactosidase, and β-glucosidase (Perea et al., 2017). *Ruminococcaceae* is an important group of microorganisms playing roles in degradation of cellulose and fermentation of plant fibers in rumen (Flint et al., 2008; Biddle et al., 2013). Consistently, the carbohydrate metabolism pathway is also higher in the rumen solid and liquid fractions than in the epithelial fraction (Table 5). These observations are consistent with the prevalence of these microorganisms in the solid and liquid fractions and suggest the role in fiber degradation.

The relative abundances of *Butyrivibrio* 2 and *Treponema* 2 were high in the epithelial fraction (Figures 2B, 3B, 4B and Table 4). *Treponema* spp. are commonly distributed in the gastrointestinal tract of ruminants, encode a wide variety of carbohydrate-active enzymes (Rosewarne et al., 2012) and act synergistically with cellulolytic bacteria to degrade cellulose and pectin to produce acetate (Liu et al., 2015). In addition, many bacteria of the genus *Butyrivibrio* produce butyrate and degrade plant fibers, such as xylans (Cotta and Forster, 2006). Acetate can accelerate rumen epithelial cell proliferation (Sakata and Tamate, 1979), and butyrate concentration is positively associated with both the absorptive surface area of the ruminal epithelium and the level of VFA oxidation in the ketogenesis pathway (Niwińska et al., 2017). Together, these results suggest a possible role for fraction specification in the determination of microbial composition.

### Changes in the Rumen Bacteria With Urea Supplementation

The PCoA and ANOSIM analyses showed significant effects of urea on the solid- associated bacterial community (Figures 2C–E, *p* ≤ 0.04), but only limited effects on the liquid-associated bacterial community (Figures 3C–E, Bray–Curtis: *p* = 0.06; unweighted UniFrac, *p* = 0.08, weighted UniFrac, *p* = 0.01); furthermore, the effects on the epithelium associated bacterial community were not significant (Figures 4C–E, *p* ≥ 0.05). Moreover, the Adonis results based on Bray-Curtis dissimilarity matrix (Figures 2C, 3C) and/or weighted Unifrac metric distance (Figures 2E, 3E), which takes bacterial abundances into account, revealed stronger discrimination in the solid- and liquid-associated bacterial communities than that based on unweighted UniFrac metric distance (Figures 2D, 3D), although the significance of the ANOSIM analysis is not strong (e.g., *p* > 0.001) because of type I error (La Rosa et al., 2012), indicating that differences in community structure (rather than community membership) distinguish among the three treatments. These results suggest that urea supplementation in the diet may differentially influence the bacteria relative abundance in the three fractions. Previous studies have demonstrated that the solid microenvironment is dominated by cellulolytic bacteria that participate in fiber degradation (Schären et al., 2017) and that rumen cellulolytic bacteria use ammonia as their sole nitrogen source (Wang and Tan, 2013). These observations may explain the significant alteration of the solid fraction by urea supplementation.

The bacteria attached to the rumen epithelium were not significantly affected by urea addition, supporting previous studies showing that the epithelial bacteria remained stable through dietary changes (Cho et al., 2006; Sadet et al., 2007). In contrast, Petri et al. (2013) and Schären et al. (2017) revealed that the rumen epithelial bacteria of dairy cattle were significantly altered during the transition from a forage diet to a high-concentrate diet (Petri et al., 2013) or a silage- and concentrate-based diet to pasture (Schären et al., 2017). In this study, the concentration of ammonia with urea supplementation differed significantly from that with the basal diet alone, but the molar concentration of total VFA did not, in contrast to the results of Schären et al. (2017). Moreover, the epithelium-attached bacteria is involved in urea hydrolysis (Costerton et al., 1979). Thus, these differences among studies in rumen fermentation parameters might explain the study differences in the response of epithelial bacteria to urea supplementation.

In light of the different effects of urea observed among the solid, liquid, and epithelial bacteria, we compared the bacterial genera among the three fractions. In all three fractions, the relative abundance of the phylum *Proteobacteria* was lower in the HU treatments than in the UC and LU treatments. This finding is consistent with previous research on the rumen microbiota of finishing bulls fed urea (Zhou et al., 2017). Members of the phylum *Proteobacteria* participate in glycine, serine, threonine and nitrogen metabolism, as revealed by a metaproteomics approach (Hart et al., 2018). Similarly, the pathway of glycine, serine and threonine metabolism is also decreased in the solid and liquid fractions (Supplementary Tables S2, S4). Therefore, these results indicate that the metabolism of glycine, serine and threonine is affected when urea is supplied into diet.

At the genus level, *Succinivibrionaceae* UCG 002 (similar to *Gilliamella* spp. based on the BLAST analysis, with 85% sequence identity) and *Ruminiclostridium* 5 were significantly increased in the three fractions with dietary urea supplementation (Figures 2F, 3F, 4F and Supplementary Tables S1, S3, S5). The *Gilliamella* phylotypes are the core bacteria in the gut of bees, and can degrade pectin, which is a compound of the pollen cell wall, and utilize mannose, arabinose, xylose, or rhamnose (Zheng et al., 2016; Praet et al., 2017). The members of *Ruminiclostridium* spp. can use cellulose, xylan, and/or cellobiose as substrates, primarily to generate acetate, ethanol and lactate (Yutin and Galperin, 2013). In rumen fermentation, the rates of degradation of highly processed grains and the hydrolysis of urea must be balanced for efficient utilization by rumen microorganisms. Interestingly, the result showed that the carbohydrate metabolism pathway also increased with dietary urea supplementation (Figures 2H, 3H). Thus, the relative increase of *Succinivibrionaceae* UCG 002 and *Ruminiclostridium* 5 in all fractions under urea supplementation is likely to relate with the increased amount of ammonia.

In the solid and liquid fractions, the relative abundance of *Oscillospira* spp. significantly increased with dietary urea supplementation (Figures 2F, 3F and Supplementary Tables S1, S3). *Oscillospira* is an enigmatic and anaerobic bacteria from *Clostridial* cluster IV that is an important butyrate producers and is associated with gut health (Gophna et al., 2017). The increase in abundance of this genus was in accordance with the increased molar concentration of butyrate (Table 2), and the increased abundance of butyrate metabolism pathway in the rumen liquid (Supplementary Table S4). In contrast, *Succinivibrionaceae* UCG 001 (similar to *Vibrio* spp. based on the BLAST analysis, with 85% sequence identity) and *Prevotella* 1 showed decreased abundance under dietary urea supplementation (Figures 2F, 3F and Supplementary Tables S1, S3). This result is consistent with previous results for finishing bulls (Zhou et al., 2017) and lambs (Ishaq et al., 2017). However, Jin et al. (2017) analyzed the *ureC* gene and found that the unclassified *Succinivibrionaceae* was enriched by urea supplementation in a RUSITEC fermenter; the difference between the present study and Jin et al. (2017) is possibly due to the study differences in the target gene (*ureC* vs. 16S rRNA) and approach (*in vitro* vs. *in vivo*).

In addition, the present study found that some bacteria within specific fractions were altered by urea supplementation. For instance, *Howardella* spp. and *Desulfobulbus* spp. were present at higher levels in the epithelial fraction of UC and LU treatments than in the HU treatment (Figure 4F and Supplementary Table S5). Previous studies revealed that the epithelial microbiota are possibly associated with oxygen consumption and urea digestion (Schären et al., 2017). *Howardella* spp. are reported to have strong ureolytic activity and to possibly play roles in urea hydrolysis (Cook et al., 2007). *Desulfobulbus* spp. participate in the reduction of sulfur compounds (Schären et al., 2017) and oxygen consumption (De Mulder et al., 2017), which are affected by the concentration of propionate (John Parkes and Graham Calder, 1985); these observations are consistent with the increased rumen concentration of propionate observed in the UC and LU treatments. These results indicate that the ureolytic and sulfur-reducing abilities of rumen bacteria may be affected when the nitrogen content is increased.

## Conclusion

In this study, we examined the effects of urea supplementation on rumen fermentation parameters and on the solid-, liquid-, and epithelium-associated bacteria. The results showed that the concentrations of ammonia, butyrate and propionate were significantly changed with dietary urea supplementation. The solid-, liquid-, and epithelium-associated bacteria are significantly different. However, the effects of urea differed among the solid, liquid, and epithelial fractions, as evidenced by the fraction differences in bacterial taxonomic composition and the predicted function. Although the differences were observed among the different fractions, our study is also limited by the results based on the 16S rRNA gene approach due to the resolution and sensitivity. Therefore, examinations of ruminal protozoa community, rumen metagenome and epithelial transcriptome are needed to further elucidate the changes in the rumen microbiota and metabolic pathways, and the rumen epithelium that occur in response to urea supplementation.

## Data Availability Statement

The datasets generated for this study can be found in the SRA database under accession number PRJNA541835.

## Ethics Statement

The animal study was reviewed and approved by Animal Care and Use Committee of Nanjing Agricultural University.

## Author Contributions

ZL, YX, and JS collected the samples. ZL and YX prepared the samples for analysis. ZL, CM, and JS analyzed the data. ZL, JS, and WZ wrote and reviewed the manuscript. JS and WZ designed the study. All authors approved the final manuscript as submitted.

## Conflict of Interest

The authors declare that the research was conducted in the absence of any commercial or financial relationships that could be construed as a potential conflict of interest.

## Supplementary Material

FIGURE S1 Box plots showing within-group similarity and between-group dissimilarity based on Bray–Curtis dissimilarity matrix in the rumen bacteria of the solid fraction under the three treatments.

FIGURE S2 Box plots showing within-group similarity and between-group dissimilarity based on Bray–Curtis dissimilarity matrix in the rumen bacteria of the liquid fraction under the three treatments.

FIGURE S3 Box plots showing within-group similarity and between-group dissimilarity based on Bray–Curtis dissimilarity matrix in the rumen bacteria of the epithelial fraction under the three treatments.

TABLE S1 The relative abundance (%) of significantly different genera in the solid fraction among the three treatments.

TABLE S2 Comparison of the predictive function in the rumen solid fraction among the three treatments.

TABLE S3 The relative abundance (%) of significantly different genera in the liquid fraction among the three treatments.

TABLE S4 Comparison of the predictive function in the rumen liquid fraction among the three treatments.

TABLE S5 The relative abundance (%) of significantly different genera in the epithelial fraction among the three treatments.

TABLE S6 Comparison of the predictive function in the rumen epithelial fraction among the three treatments.

## Abbreviations

ADG: average daily gain
ANOSIM: analysis of similarities
DM: dry matter
DMI: dry matter intake
NPN: non-protein nitrogen
OTU: operational taxonomic unit
PCoA: principal coordinates analysis
PICRUSt: phylogenetic investigation of communities by reconstruction of unobserved states
SD: standard deviation
TMR: total mixed ration
VFA: volatile fatty acid.
