# Supplementary figures and images for: Changes in the Solid-, Liquid-, and Epithelium-Associated Bacterial Communities in the Rumen of Hu Lambs in Response to Dietary Urea Supplementation
Source: Front Microbiol. 2020 Feb 21;11:244. doi: 10.3389/fmicb.2020.00244 (PMC7046558; doi:10.3389/fmicb.2020.00244)

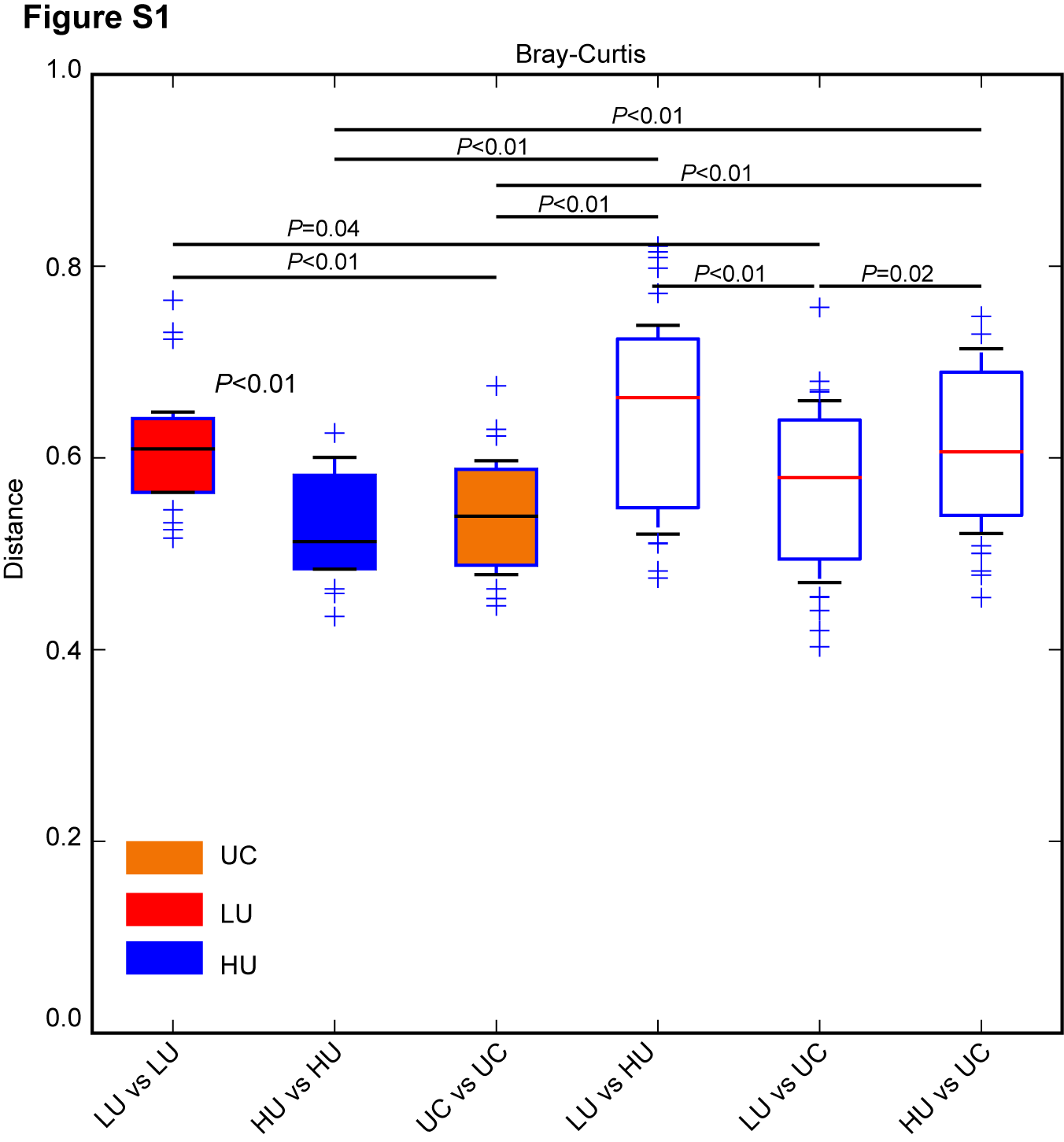

Supplement: FIGURE S1 — Box plots showing within-group similarity and between-group dissimilarity based on Bray–Curtis dissimilarity matrix in the rumen bacteria of the solid fraction under the three treatments. [file Image_1.TIF]

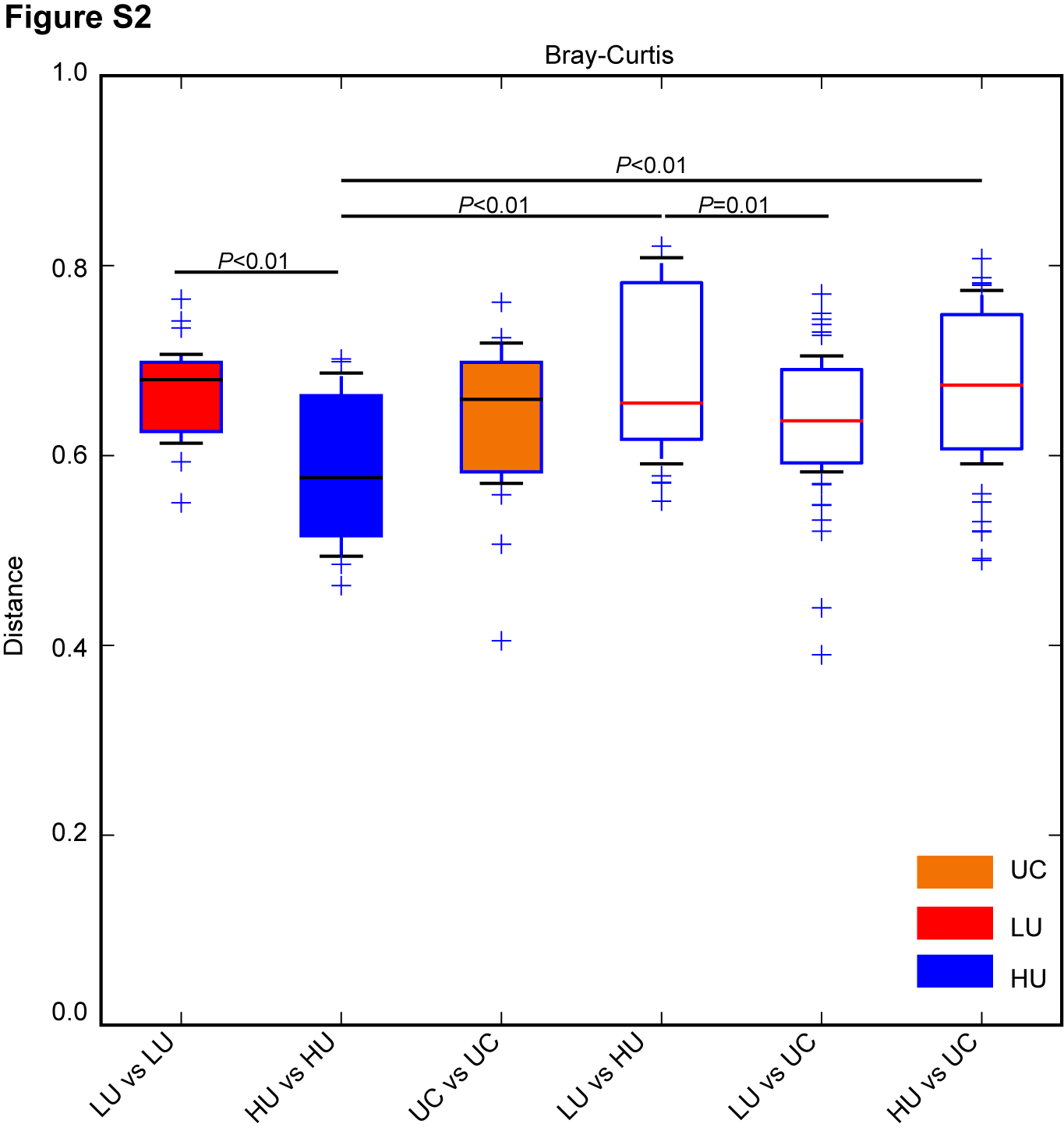

Supplement: FIGURE S2 — Box plots showing within-group similarity and between-group dissimilarity based on Bray–Curtis dissimilarity matrix in the rumen bacteria of the liquid fraction under the three treatments. [file Image_2.TIF]

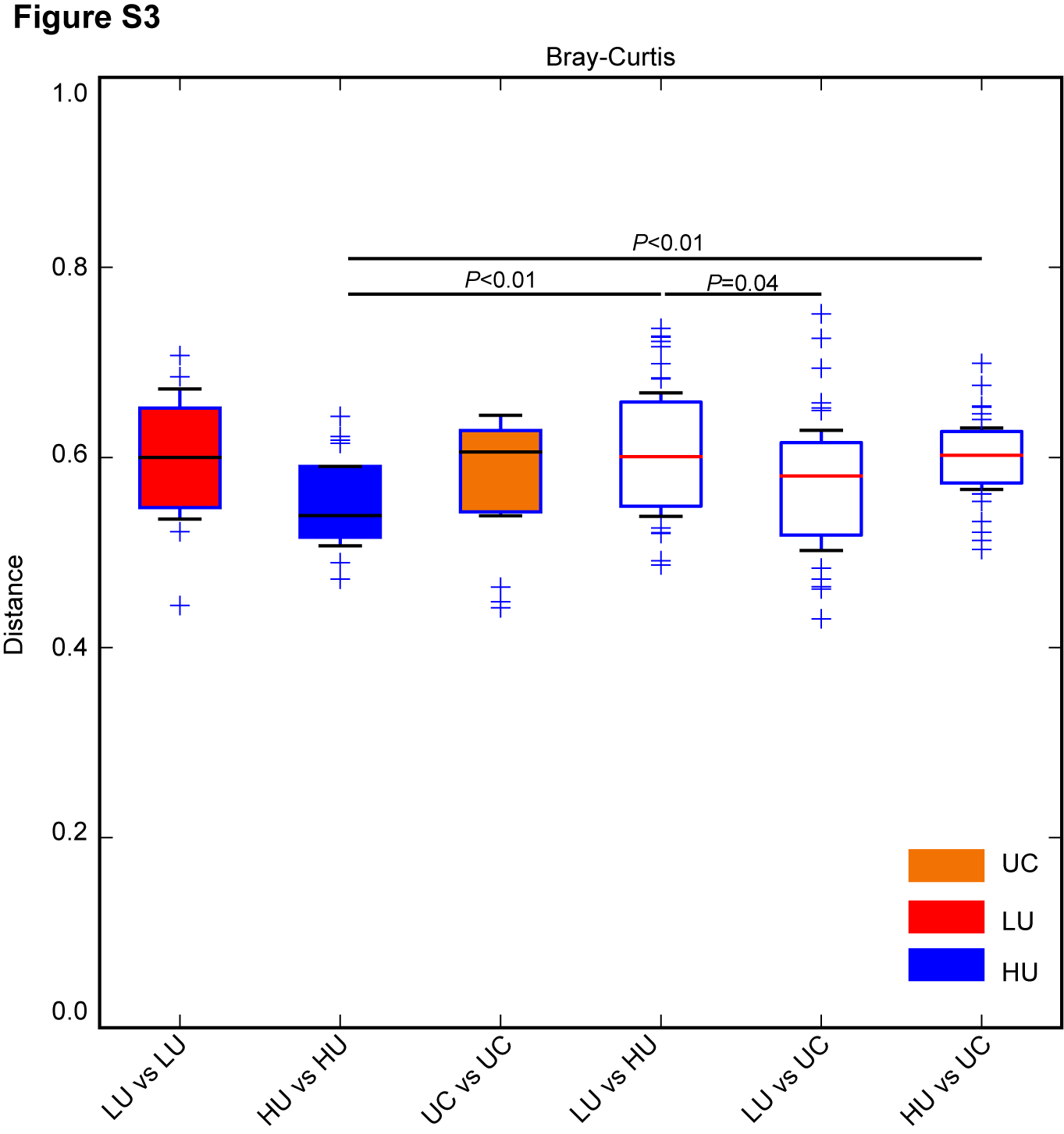

Supplement: FIGURE S3 — Box plots showing within-group similarity and between-group dissimilarity based on Bray–Curtis dissimilarity matrix in the rumen bacteria of the epithelial fraction under the three treatments. [file Image_3.TIF]
