# Supplementary material for: Changes in the Solid-, Liquid-, and Epithelium-Associated Bacterial Communities in the Rumen of Hu Lambs in Response to Dietary Urea Supplementation
Source: Front Microbiol. 2020 Feb 21;11:244. doi: 10.3389/fmicb.2020.00244 (PMC7046558; doi:10.3389/fmicb.2020.00244)
Supplement: TABLE S1 — The relative abundance (%) of significantly different genera in the solid fraction among the three treatments. [file Table_1.DOCX]

**Table S1**. The relative abundance (%) of significantly different genera in the solid fraction among the three treatments.

| Genus | Mean | | |
| --- | --- | --- | --- |
|  | UC | LU | HU |
| Prevotella 1 | 19.88 | 24.12 | 12.38 |
| Succinivibrionaceae UCG 001 | 14.45 | 14.41 | 0.90 |
| Christensenellaceae R7 | 4.98 | 3.92 | 6.21 |
| Succiniclasticum | 4.77 | 4.12 | 2.33 |
| Rikenellaceae RC9 | 3.14 | 2.79 | 6.79 |
| Butyrivibrio 2 | 0.72 | 0.64 | 1.07 |
| Lachnospiraceae uncultured | 0.68 | 0.71 | 3.07 |
| Ruminococcus gauvreauii | 0.60 | 0.52 | 0.32 |
| Lachnospiraceae AC2044 | 0.58 | 0.71 | 1.04 |
| Succinivibrionaceae UCG 002 | 0.47 | 0.87 | 3.31 |
| Ruminococcaceae UCG 010 | 0.38 | 0.28 | 1.02 |
| Prevotella 7 | 0.28 | 0.15 | 0.02 |
| Anaerovorax | 0.23 | 0.23 | 0.45 |
| Pseudobutyrivibrio | 0.22 | 0.24 | 0.57 |
| Blautia | 0.20 | 0.12 | 0.18 |
| Marvinbryantia | 0.17 | 0.14 | 0.25 |
| Lachnoclostridium 10 | 0.16 | 0.10 | 0.28 |
| Desulfovibrio | 0.15 | 0.12 | 0.28 |
| Ruminobacter | 0.14 | 0.35 | 2.78 |
| Olsenella | 0.10 | 0.16 | 0.02 |
| Succinimonas | 0.10 | 0.02 | 0.50 |
| Roseburia | 0.07 | 0.05 | 0.10 |
| Veillonellaceae uncultured | 0.07 | 0.06 | 0.02 |
| Erysipelotrichaceae uncultured | 0.07 | 0.04 | 0.09 |
| Rhodospirillales norank | 0.06 | 0.07 | 0.21 |
| Selenomonas 3 | 0.05 | 0.01 | 0.01 |
| Ruminococcus torques | 0.05 | 0.01 | 0.02 |
| Atopobium | 0.04 | 0.06 | 0.02 |
| Lachnospira | 0.04 | 0.03 | 0.01 |
| Ruminiclostridium 6 | 0.03 | 0.04 | 0.12 |
| Family XIII uncultured | 0.02 | 0.02 | 0.04 |
| Anaerofustis | 0.01 | 0.01 | 0.03 |
| Clostridiales vadinBB60 | 0.01 | 0.01 | 0.04 |
| Bacteroidetes BD2 2 | 0.01 | 0.03 | 0.16 |
| Ruminococcaceae V9D2013 | 0.01 | 0.02 | 0.13 |
| Oscillospira | 0.00 | 0.00 | 0.01 |
| Ruminiclostridium 5 | 0.00 | 0.01 | 0.11 |
