# Supplementary material for: Changes in the Solid-, Liquid-, and Epithelium-Associated Bacterial Communities in the Rumen of Hu Lambs in Response to Dietary Urea Supplementation
Source: Front Microbiol. 2020 Feb 21;11:244. doi: 10.3389/fmicb.2020.00244 (PMC7046558; doi:10.3389/fmicb.2020.00244)
Supplement: TABLE S2 — Comparison of the predictive function in the rumen solid fraction among the three treatments. [file Table_2.DOCX]

**Table S2**. Comparison of the predictive function in the rumen solid fraction among the three treatments

| KEGG level 3 | Mean (%) | | | *p* value | | |
| --- | --- | --- | --- | --- | --- | --- |
|  | UC | LU | HU | UC VS LU | UC VS HU | LU VS HU |
| Alanine aspartate and glutamate metabolism | 1.14 | 1.15 | 1.12 | 0.39 | 0.06 | 0.04 |
| Amino acid related enzymes | 1.59 | 1.60 | 1.57 | 0.82 | 0.04 | 0.02 |
| Amoebiasis | 0.01 | 0.01 | 0.01 | 0.63 | 0.06 | 0.03 |
| Amyotrophic lateral sclerosis | 0.01 | 0.01 | 0.01 | 0.59 | 0.01 | 0.03 |
| Arachidonic acid metabolism | 0.04 | 0.04 | 0.03 | 0.82 | 0.03 | 0.03 |
| Atrazine degradation | 0.01 | 0.01 | 0.01 | 0.18 | 0.09 | 0.01 |
| Bacterial chemotaxis | 0.43 | 0.42 | 0.53 | 0.70 | 0.00 | 0.00 |
| Bacterial motility proteins | 0.89 | 0.86 | 1.09 | 0.70 | 0.00 | 0.00 |
| Base excision repair | 0.47 | 0.46 | 0.45 | 0.39 | 0.01 | 0.09 |
| Biosynthesis and biodegradation of secondary metabolites | 0.06 | 0.06 | 0.06 | 0.24 | 0.13 | 0.04 |
| Biosynthesis of ansamycins | 0.10 | 0.10 | 0.11 | 0.48 | 0.03 | 0.04 |
| Biosynthesis of siderophore group nonribosomal peptides | 0.02 | 0.02 | 0.01 | 0.39 | 0.13 | 0.02 |
| Butirosin and neomycin biosynthesis | 0.07 | 0.08 | 0.08 | 0.59 | 0.02 | 0.18 |
| Calcium signaling pathway | 0.00 | 0.00 | 0.00 | 0.31 | 0.00 | 0.04 |
| Carbon fixation pathways in prokaryotes | 1.11 | 1.10 | 1.07 | 0.48 | 0.02 | 0.09 |
| Cellular antigens | 0.05 | 0.05 | 0.04 | 0.82 | 0.06 | 0.04 |
| Chaperones and folding catalysts | 1.08 | 1.10 | 1.04 | 0.59 | 0.06 | 0.00 |
| Chloroalkane and chloroalkene degradation | 0.17 | 0.16 | 0.18 | 0.59 | 0.09 | 0.03 |
| Citrate cycle | 0.72 | 0.72 | 0.69 | 0.94 | 0.03 | 0.00 |
| DNA repair and recombination proteins | 3.08 | 3.10 | 3.02 | 0.48 | 0.06 | 0.03 |
| DNA replication | 0.73 | 0.73 | 0.71 | 0.70 | 0.09 | 0.03 |
| DNA replication proteins | 1.36 | 1.36 | 1.33 | 0.70 | 0.04 | 0.03 |
| Ether lipid metabolism | 0.00 | 0.00 | 0.00 | 0.24 | 0.20 | 0.04 |
| Flagellar assembly | 0.35 | 0.34 | 0.46 | 0.82 | 0.01 | 0.01 |
| Flavonoid biosynthesis | 0.01 | 0.01 | 0.01 | 0.94 | 0.03 | 0.18 |
| Folate biosynthesis | 0.42 | 0.43 | 0.40 | 1.00 | 0.02 | 0.00 |
| Function unknown | 1.31 | 1.32 | 1.26 | 0.94 | 0.04 | 0.24 |
| Galactose metabolism | 0.63 | 0.64 | 0.68 | 1.00 | 0.02 | 0.18 |
| Geraniol degradation | 0.06 | 0.07 | 0.05 | 0.82 | 0.18 | 0.04 |
| Germination | 0.03 | 0.03 | 0.04 | 0.82 | 0.06 | 0.04 |
| Glutathione metabolism | 0.22 | 0.22 | 0.18 | 0.82 | 0.04 | 0.13 |
| Glycerolipid metabolism | 0.33 | 0.33 | 0.37 | 1.00 | 0.02 | 0.01 |
| Glycine serine and threonine metabolism | 0.89 | 0.91 | 0.87 | 0.39 | 0.13 | 0.02 |
| Glycosyltransferases | 0.37 | 0.38 | 0.34 | 0.70 | 0.04 | 0.04 |
| Glyoxylate and dicarboxylate metabolism | 0.52 | 0.53 | 0.51 | 0.70 | 0.04 | 0.03 |
| Huntington's disease | 0.03 | 0.03 | 0.03 | 0.94 | 0.06 | 0.03 |
| Insulin signaling pathway | 0.08 | 0.08 | 0.09 | 0.94 | 0.03 | 0.24 |
| Ion channels | 0.01 | 0.01 | 0.01 | 0.70 | 0.03 | 0.18 |
| Isoquinoline alkaloid biosynthesis | 0.06 | 0.06 | 0.05 | 1.00 | 0.02 | 0.00 |
| Lipopolysaccharide biosynthesis | 0.38 | 0.39 | 0.29 | 0.94 | 0.04 | 0.01 |
| Lipopolysaccharide biosynthesis proteins | 0.49 | 0.50 | 0.39 | 0.94 | 0.04 | 0.03 |
| Lysine biosynthesis | 0.86 | 0.85 | 0.84 | 0.70 | 0.03 | 0.03 |
| Lysosome | 0.09 | 0.09 | 0.11 | 0.94 | 0.02 | 0.24 |
| Membrane and intracellular structural molecules | 0.66 | 0.69 | 0.58 | 0.59 | 0.06 | 0.00 |
| Metabolism of cofactors and vitamins | 0.11 | 0.11 | 0.09 | 0.94 | 0.01 | 0.02 |
| Nicotinate and nicotinamide metabolism | 0.49 | 0.51 | 0.47 | 0.39 | 0.01 | 0.00 |
| One carbon pool by folate | 0.71 | 0.72 | 0.69 | 0.70 | 0.09 | 0.04 |
| Others | 0.77 | 0.77 | 0.80 | 0.82 | 0.03 | 0.13 |
| Oxidative phosphorylation | 1.27 | 1.26 | 1.21 | 0.82 | 0.02 | 0.13 |
| Pantothenate and CoA biosynthesis | 0.70 | 0.69 | 0.68 | 0.70 | 0.04 | 0.04 |
| Phenylalanine tyrosine and tryptophan biosynthesis | 0.98 | 0.99 | 0.96 | 0.39 | 0.03 | 0.04 |
| Phosphonate and phosphinate metabolism | 0.06 | 0.06 | 0.06 | 0.59 | 0.03 | 0.13 |
| Photosynthesis - antenna proteins | 0.00 | 0.00 | 0.00 | 0.30 | 0.00 | 0.04 |
| Plant-pathogen interaction | 0.15 | 0.15 | 0.16 | 0.59 | 0.00 | 0.00 |
| Pores ion channels | 0.37 | 0.38 | 0.33 | 0.82 | 0.09 | 0.03 |
| Prenyltransferases | 0.36 | 0.37 | 0.34 | 1.00 | 0.03 | 0.00 |
| Primary bile acid biosynthesis | 0.02 | 0.02 | 0.03 | 1.00 | 0.03 | 0.06 |
| Primary immunodeficiency | 0.04 | 0.04 | 0.04 | 0.48 | 0.48 | 0.04 |
| Propanoate metabolism | 0.50 | 0.49 | 0.50 | 0.04 | 0.82 | 0.24 |
| Proteasome | 0.05 | 0.05 | 0.05 | 0.82 | 0.06 | 0.03 |
| Protein digestion and absorption | 0.03 | 0.03 | 0.02 | 0.59 | 0.09 | 0.04 |
| Protein export | 0.67 | 0.67 | 0.66 | 0.82 | 0.13 | 0.04 |
| Protein kinases | 0.24 | 0.23 | 0.27 | 0.70 | 0.04 | 0.01 |
| Purine metabolism | 2.35 | 2.36 | 2.28 | 0.70 | 0.02 | 0.00 |
| Riboflavin metabolism | 0.27 | 0.27 | 0.24 | 0.94 | 0.04 | 0.04 |
| Ribosome | 2.70 | 2.71 | 2.63 | 0.59 | 0.02 | 0.03 |
| Ribosome biogenesis in eukaryotes | 0.07 | 0.07 | 0.06 | 1.00 | 0.00 | 0.03 |
| RNA degradation | 0.52 | 0.52 | 0.49 | 1.00 | 0.06 | 0.02 |
| RNA polymerase | 0.20 | 0.19 | 0.19 | 0.31 | 0.00 | 0.06 |
| Secondary bile acid biosynthesis | 0.02 | 0.02 | 0.02 | 1.00 | 0.04 | 0.06 |
| Sporulation | 0.52 | 0.50 | 0.67 | 0.94 | 0.02 | 0.03 |
| Toluene degradation | 0.11 | 0.11 | 0.09 | 0.82 | 0.06 | 0.02 |
| Transcription factors | 1.33 | 1.32 | 1.44 | 0.82 | 0.04 | 0.02 |
| Translation proteins | 1.02 | 1.02 | 0.99 | 0.70 | 0.00 | 0.01 |
| Transporters | 5.25 | 5.16 | 5.74 | 0.94 | 0.02 | 0.00 |
| Tropane piperidine and pyridine alkaloid biosynthesis | 0.13 | 0.13 | 0.12 | 0.70 | 0.06 | 0.04 |
| Two-component system | 1.28 | 1.26 | 1.39 | 0.48 | 0.00 | 0.00 |
| Ubiquinone and other terpenoid-quinone biosynthesis | 0.26 | 0.28 | 0.20 | 1.00 | 0.06 | 0.00 |
| Vitamin B6 metabolism | 0.24 | 0.24 | 0.22 | 0.39 | 0.09 | 0.04 |
| Xylene degradation | 0.04 | 0.04 | 0.05 | 0.94 | 0.06 | 0.04 |
| Zeatin biosynthesis | 0.06 | 0.06 | 0.06 | 0.39 | 0.18 | 0.04 |
