# Supplementary material for: Changes in the Solid-, Liquid-, and Epithelium-Associated Bacterial Communities in the Rumen of Hu Lambs in Response to Dietary Urea Supplementation
Source: Front Microbiol. 2020 Feb 21;11:244. doi: 10.3389/fmicb.2020.00244 (PMC7046558; doi:10.3389/fmicb.2020.00244)
Supplement: TABLE S3 — The relative abundance (%) of significantly different genera in the liquid fraction among the three treatments. [file Table_3.DOCX]

**Table S3**. The relative abundance (%) of significantly different genera in the liquid fraction among the three treatments

| Genus | Mean | | |
| --- | --- | --- | --- |
|  | UC | LU | HU |
| Prevotella 1 | 24.48 | 21.37 | 12.12 |
| Succinivibrionaceae UCG 001 | 14.21 | 10.07 | 0.18 |
| Rikenellaceae RC9 | 3.78 | 3.65 | 6.77 |
| Ruminococcaceae NK4A214 | 2.60 | 2.60 | 4.73 |
| Succiniclasticum | 2.42 | 1.52 | 1.31 |
| Prevotellaceae UCG 003 | 2.40 | 1.45 | 1.54 |
| Ruminococcus 2 | 1.15 | 2.53 | 2.22 |
| Veillonellaceae UCG 001 | 0.78 | 0.37 | 0.82 |
| Eubacterium coprostanoligenes | 0.62 | 0.47 | 1.26 |
| Prevotellaceae NK3B31 | 0.52 | 0.21 | 0.61 |
| Ruminococcaceae UCG 010 | 0.37 | 0.40 | 0.95 |
| Prevotellaceae YAB2003 | 0.35 | 0.34 | 0.09 |
| Prevotellaceae UCG 004 | 0.32 | 0.17 | 0.30 |
| Selenomonas 3 | 0.28 | 0.06 | 0.11 |
| Succinivibrionaceae UCG 002 | 0.22 | 1.35 | 1.85 |
| Anaerovorax | 0.15 | 0.12 | 0.26 |
| Pseudobutyrivibrio | 0.13 | 0.19 | 0.26 |
| Papillibacter | 0.08 | 0.07 | 0.23 |
| Lachnoclostridium 1 | 0.07 | 0.09 | 0.03 |
| Ruminiclostridium 9 | 0.06 | 0.02 | 0.15 |
| Eubacterium nodatum | 0.05 | 0.07 | 0.10 |
| Lachnoclostridium 10 | 0.04 | 0.03 | 0.07 |
| Marvinbryantia | 0.04 | 0.06 | 0.22 |
| Ruminiclostridium 6 | 0.04 | 0.04 | 0.11 |
| Lachnospiraceae FCS020 | 0.04 | 0.04 | 0.09 |
| Howardella | 0.04 | 0.03 | 0.01 |
| Succinimonas | 0.03 | 0.05 | 0.27 |
| Lachnospira | 0.03 | 0.05 | 0.01 |
| Ruminococcaceae UCG 004 | 0.02 | 0.02 | 0.04 |
| Desulfobulbus | 0.02 | 0.04 | 0.02 |
| Ruminococcaceae V9D2013 | 0.01 | 0.04 | 0.12 |
| Eubacterium brachy | 0.01 | 0.02 | 0.06 |
| Family XIII UCG 001 | 0.01 | 0.01 | 0.02 |
| Coprococcus 2 | 0.00 | 0.02 | 0.17 |
| Oscillospira | 0.00 | 0.03 | 0.06 |
| Eubacterium cellulosolvens | 0.00 | 0.06 | 0.01 |
| Ruminiclostridium 5 | 0.00 | 0.00 | 0.04 |
