# Supplementary material for: Changes in the Solid-, Liquid-, and Epithelium-Associated Bacterial Communities in the Rumen of Hu Lambs in Response to Dietary Urea Supplementation
Source: Front Microbiol. 2020 Feb 21;11:244. doi: 10.3389/fmicb.2020.00244 (PMC7046558; doi:10.3389/fmicb.2020.00244)
Supplement: TABLE S4 — Comparison of the predictive function in the rumen liquid fraction among the three treatments. [file Table_4.DOCX]

**Table S4**. Comparison of the predictive function in the rumen liquid fraction among the three treatments

| KEGG level3 | Mean (%) | | | *p* value | | |
| --- | --- | --- | --- | --- | --- | --- |
|  | UC | LU | HU | UC VS LU | UC VS HU | LU VS HU |
| Amino acid related enzymes | 0.01 | 0.01 | 0.01 | 0.03 | 0.70 | 0.13 |
| Flagellar assembly | 0.2 | 0.19 | 0.19 | 0.04 | 1.00 | 0.31 |
| Flavonoid biosynthesis | 0.64 | 0.62 | 0.64 | 0.03 | 0.59 | 0.03 |
| Folate biosynthesis | 1.96 | 1.97 | 1.92 | 0.39 | 0.04 | 0.00 |
| Galactose metabolism | 0.02 | 0.02 | 0.02 | 0.39 | 0.31 | 0.00 |
| Geraniol degradation | 1.16 | 1.17 | 1.12 | 0.70 | 0.04 | 0.00 |
| Germination | 0.33 | 0.33 | 0.35 | 0.82 | 0.04 | 0.00 |
| Glutathione metabolism | 0.01 | 0.01 | 0.01 | 0.82 | 0.01 | 0.01 |
| Glycan biosynthesis and metabolism | 0.19 | 0.18 | 0.21 | 0.59 | 0.03 | 0.01 |
| Glycerolipid metabolism | 0.25 | 0.25 | 0.23 | 0.94 | 0.00 | 0.02 |
| Glycine serine and threonine metabolism | 1.13 | 1.12 | 1.03 | 1.00 | 0.00 | 0.02 |
| Glycolysis / Gluconeogenesis | 0.04 | 0.04 | 0.04 | 0.82 | 0.02 | 0.02 |
| Glycosyltransferases | 1.07 | 1.07 | 1.02 | 1.00 | 0.03 | 0.02 |
| Amoebiasis | 0.91 | 0.91 | 0.87 | 0.82 | 0.04 | 0.02 |
| Hepatitis C | 1.57 | 1.57 | 1.49 | 0.59 | 0.06 | 0.02 |
| Homologous recombination | 1.71 | 1.72 | 1.62 | 0.82 | 0.06 | 0.02 |
| Huntington's disease | 0.41 | 0.39 | 0.31 | 0.70 | 0.00 | 0.03 |
| Insulin signaling pathway | 0.92 | 0.92 | 0.88 | 0.82 | 0.00 | 0.03 |
| Ion channels | 0.53 | 0.52 | 0.48 | 0.59 | 0.01 | 0.03 |
| Isoquinoline alkaloid biosynthesis | 0.16 | 0.16 | 0.19 | 0.82 | 0.01 | 0.03 |
| Lipopolysaccharide biosynthesis | 0.05 | 0.04 | 0.02 | 0.94 | 0.01 | 0.03 |
| Lipopolysaccharide biosynthesis proteins | 1.15 | 1.15 | 1.04 | 0.70 | 0.02 | 0.03 |
| Lysine degradation | 0 | 0 | 0.01 | 0.82 | 0.02 | 0.03 |
| Measles | 0.35 | 0.36 | 0.35 | 0.59 | 0.24 | 0.03 |
| Membrane and intracellular structural molecules | 0.01 | 0.01 | 0.01 | 0.70 | 0.24 | 0.03 |
| Methane metabolism | 0.45 | 0.43 | 0.39 | 0.59 | 0.00 | 0.04 |
| Mismatch repair | 0.3 | 0.29 | 0.2 | 0.70 | 0.00 | 0.04 |
| Arachidonic acid metabolism | 0.74 | 0.71 | 0.56 | 1.00 | 0.00 | 0.04 |
| Nicotinate and nicotinamide metabolism | 1.33 | 1.29 | 1.17 | 0.48 | 0.00 | 0.04 |
| One carbon pool by folate | 0.39 | 0.38 | 0.33 | 0.59 | 0.01 | 0.04 |
| Other ion-coupled transporters | 1.22 | 1.28 | 1.46 | 0.59 | 0.01 | 0.04 |
| Others | 0.03 | 0.03 | 0.04 | 0.70 | 0.01 | 0.04 |
| Oxidative phosphorylation | 0.22 | 0.22 | 0.17 | 0.70 | 0.01 | 0.04 |
| Pathways in cancer | 0.07 | 0.06 | 0.04 | 0.94 | 0.01 | 0.04 |
| Peptidases | 0.03 | 0.03 | 0.02 | 0.94 | 0.01 | 0.04 |
| Peptidoglycan biosynthesis | 0.22 | 0.23 | 0.27 | 1.00 | 0.01 | 0.04 |
| Pertussis | 1.37 | 1.33 | 1.52 | 0.94 | 0.04 | 0.04 |
| Phagosome | 0.09 | 0.09 | 0.08 | 0.82 | 0.13 | 0.04 |
| Photosynthesis | 0.52 | 0.52 | 0.5 | 0.70 | 0.18 | 0.04 |
| Bacterial invasion of epithelial cells | 0.01 | 0.01 | 0 | 0.94 | 0.09 | 0.04 |
| Protein digestion and absorption | 0.44 | 0.41 | 0.28 | 0.70 | 0.00 | 0.06 |
| Protein kinases | 0.55 | 0.51 | 0.37 | 0.70 | 0.00 | 0.06 |
| Purine metabolism | 0.12 | 0.11 | 0.09 | 0.94 | 0.00 | 0.06 |
| Pyruvate metabolism | 0.33 | 0.34 | 0.39 | 1.00 | 0.00 | 0.06 |
| Bacterial motility proteins | 0.73 | 0.72 | 0.67 | 0.94 | 0.00 | 0.06 |
| RNA degradation | 0.36 | 0.4 | 0.49 | 0.48 | 0.01 | 0.06 |
| RNA polymerase | 0.76 | 0.81 | 1.03 | 0.70 | 0.01 | 0.06 |
| Riboflavin metabolism | 0.74 | 0.74 | 0.71 | 1.00 | 0.01 | 0.06 |
| Ribosome Biogenesis | 0.29 | 0.32 | 0.45 | 0.82 | 0.02 | 0.06 |
| Secondary bile acid biosynthesis | 0.37 | 0.37 | 0.32 | 0.82 | 0.02 | 0.06 |
| Selenocompound metabolism | 0.37 | 0.37 | 0.33 | 0.82 | 0.02 | 0.06 |
| Bacterial toxins | 0.06 | 0.06 | 0.06 | 0.94 | 0.02 | 0.06 |
| Sporulation | 0.91 | 0.9 | 0.88 | 1.00 | 0.04 | 0.06 |
| Styrene degradation | 0.28 | 0.27 | 0.22 | 0.70 | 0.00 | 0.09 |
| Synthesis and degradation of ketone bodies | 4.75 | 4.98 | 5.75 | 0.70 | 0.00 | 0.09 |
| Taurine and hypotaurine metabolism | 0.01 | 0.01 | 0 | 0.48 | 0.02 | 0.09 |
| Thiamine metabolism | 0.75 | 0.78 | 0.81 | 0.59 | 0.03 | 0.09 |
| Toluene degradation | 0.57 | 0.56 | 0.54 | 0.70 | 0.03 | 0.09 |
| Transcription factors | 0.06 | 0.06 | 0.05 | 1.00 | 0.03 | 0.09 |
| Benzoate degradation | 1.61 | 1.6 | 1.56 | 0.59 | 0.04 | 0.09 |
| Transporters | 0.52 | 0.51 | 0.49 | 0.94 | 0.04 | 0.09 |
| Tropane piperidine and pyridine alkaloid biosynthesis | 0.07 | 0.06 | 0.04 | 0.59 | 0.00 | 0.13 |
| Two-component system | 0.38 | 0.37 | 0.34 | 0.59 | 0.00 | 0.13 |
| Ubiquinone and other terpenoid-quinone biosynthesis | 1.19 | 1.23 | 1.33 | 0.48 | 0.01 | 0.13 |
| Valine leucine and isoleucine degradation | 0.44 | 0.51 | 0.68 | 0.59 | 0.01 | 0.13 |
| Vibrio cholerae infection | 0.08 | 0.09 | 0.11 | 0.82 | 0.01 | 0.13 |
| Vitamin B6 metabolism | 0.04 | 0.04 | 0.05 | 0.94 | 0.01 | 0.13 |
| Bile secretion | 0.01 | 0.01 | 0.01 | 0.82 | 0.02 | 0.13 |
| Xylene degradation | 3.13 | 3.12 | 3.03 | 0.70 | 0.04 | 0.13 |
| Zeatin biosynthesis | 1 | 1.01 | 1.06 | 1.00 | 0.04 | 0.13 |
| alpha-Linolenic acid metabolism | 0.23 | 0.22 | 0.19 | 0.70 | 0.01 | 0.18 |
| mRNA surveillance pathway | 0.04 | 0.04 | 0.05 | 1.00 | 0.01 | 0.18 |
| mTOR signaling pathway | 0.06 | 0.06 | 0.05 | 0.39 | 0.00 | 0.24 |
| Biosynthesis of ansamycins | 0.06 | 0.06 | 0.05 | 0.70 | 0.02 | 0.24 |
| Biosynthesis of siderophore group nonribosomal peptides | 0.12 | 0.12 | 0.11 | 0.82 | 0.03 | 0.24 |
| Biosynthesis of unsaturated fatty acids | 0.08 | 0.08 | 0.09 | 0.48 | 0.04 | 0.24 |
| Butanoate metabolism | 0.15 | 0.15 | 0.16 | 0.24 | 0.02 | 0.31 |
| C5-Branched dibasic acid metabolism | 1.07 | 1.09 | 1.13 | 0.94 | 0.02 | 0.31 |
| Caprolactam degradation | 0.34 | 0.36 | 0.38 | 0.82 | 0.04 | 0.31 |
| Cell cycle | 2.39 | 2.36 | 2.3 | 0.31 | 0.00 | 0.39 |
| Cell cycle - Caulobacter | 0.02 | 0.01 | 0.01 | 1.00 | 0.02 | 0.39 |
| Alanine aspartate and glutamate metabolism | 0.13 | 0.12 | 0.12 | 0.48 | 0.04 | 0.39 |
| Cell division | 0.04 | 0.04 | 0.03 | 0.59 | 0.01 | 0.48 |
| Cell motility and secretion | 0.02 | 0.02 | 0.03 | 0.70 | 0.03 | 0.48 |
| Cellular antigens | 0.09 | 0.1 | 0.1 | 0.39 | 0.04 | 0.48 |
| Chaperones and folding catalysts | 0.65 | 0.68 | 0.72 | 0.39 | 0.04 | 0.48 |
| Chloroalkane and chloroalkene degradation | 0.03 | 0.02 | 0.02 | 0.48 | 0.01 | 0.59 |
| Chromosome | 0.04 | 0.03 | 0.03 | 0.94 | 0.01 | 0.59 |
| Cytoskeleton proteins | 0.02 | 0.02 | 0.03 | 0.70 | 0.03 | 0.70 |
| DNA repair and recombination proteins | 0.13 | 0.13 | 0.12 | 0.70 | 0.04 | 0.70 |
| DNA replication | 0.14 | 0.13 | 0.12 | 0.24 | 0.00 | 0.82 |
| Dioxin degradation | 0.26 | 0.23 | 0.23 | 0.48 | 0.03 | 0.94 |
