# Supplementary material for: Changes in the Solid-, Liquid-, and Epithelium-Associated Bacterial Communities in the Rumen of Hu Lambs in Response to Dietary Urea Supplementation
Source: Front Microbiol. 2020 Feb 21;11:244. doi: 10.3389/fmicb.2020.00244 (PMC7046558; doi:10.3389/fmicb.2020.00244)
Supplement: TABLE S5 — The relative abundance (%) of significantly different genera in the epithelial fraction among the three treatments. [file Table_5.DOCX]

**Table S5**. The relative abundance (%) of significantly different genera in the epithelial fraction among the three treatments

| Genus | Mean | | |
| --- | --- | --- | --- |
|  | UC | LU | HU |
| Prevotellaceae UCG 001 | 7.99 | 4.73 | 9.21 |
| Treponema 2 | 4.95 | 4.67 | 7.94 |
| Desulfobulbus | 3.53 | 3.45 | 1.57 |
| Christensenellaceae R7 | 3.27 | 2.01 | 2.82 |
| Fibrobacter | 1.43 | 1.66 | 2.74 |
| Prevotellaceae UCG 003 | 0.76 | 1.05 | 1.63 |
| Howardella | 0.67 | 0.40 | 0.17 |
| Prevotellaceae NK3B31 | 0.49 | 0.34 | 0.68 |
| Defluviitaleaceae UCG 011 | 0.41 | 0.30 | 0.12 |
| Ruminococcaceae UCG 010 | 0.29 | 0.24 | 0.56 |
| Prevotellaceae UCG 004 | 0.28 | 0.31 | 0.56 |
| Succinivibrionaceae UCG 002 | 0.27 | 0.93 | 2.45 |
| Lachnospiraceae UCG 010 | 0.21 | 0.11 | 0.12 |
| Ruminococcaceae uncultured | 0.18 | 0.18 | 0.41 |
| Ruminobacter | 0.16 | 0.89 | 1.83 |
| Lachnospiraceae AC2044 | 0.10 | 0.18 | 0.27 |
| Papillibacter | 0.10 | 0.05 | 0.21 |
| Olsenella | 0.09 | 0.18 | 0.06 |
| Eubacterium hallii | 0.06 | 0.05 | 0.07 |
| Lachnoclostridium 10 | 0.05 | 0.05 | 0.13 |
| Lachnospiraceae XPB1014 | 0.05 | 0.09 | 0.19 |
| Clostridiales vadinBB60 | 0.03 | 0.04 | 0.08 |
| Marvinbryantia | 0.03 | 0.02 | 0.05 |
| Succinimonas | 0.03 | 0.04 | 0.46 |
| Lachnospiraceae FCS020 | 0.02 | 0.03 | 0.06 |
| Ruminiclostridium 6 | 0.01 | 0.01 | 0.03 |
| Ruminococcaceae UCG 013 | 0.01 | 0.00 | 0.01 |
| Oscillospira | 0.00 | 0.02 | 0.04 |
| Ruminiclostridium 5 | 0.00 | 0.00 | 0.04 |
