# Supplementary material for: Changes in the Solid-, Liquid-, and Epithelium-Associated Bacterial Communities in the Rumen of Hu Lambs in Response to Dietary Urea Supplementation
Source: Front Microbiol. 2020 Feb 21;11:244. doi: 10.3389/fmicb.2020.00244 (PMC7046558; doi:10.3389/fmicb.2020.00244)
Supplement: TABLE S6 — Comparison of the predictive function in the rumen epithelial fraction among the three treatments. [file Table_6.DOCX]

**Table S6**. Comparison of the predictive function in the rumen epithelial fraction among the three treatments

| KEGG level 3 | Mean (%) | | | *p* value | | |
| --- | --- | --- | --- | --- | --- | --- |
|  | UC | LU | HU | UC VS LU | UC VS HU | LU VS HU |
| Galactose metabolism | 0.08 | 0.08 | 0.06 | 0.70 | 0.09 | 0.04 |
| Glutamatergic synapse | 0.02 | 0.02 | 0.02 | 0.82 | 0.06 | 0.04 |
| Glycan biosynthesis and metabolism | 0.98 | 0.97 | 0.99 | 0.31 | 0.18 | 0.04 |
| Glycine serine and threonine metabolism | 0.52 | 0.5 | 0.51 | 0.06 | 0.48 | 0.04 |
| Insulin signaling pathway | 0.01 | 0.01 | 0.01 | 0.18 | 0.82 | 0.03 |
| Isoflavonoid biosynthesis | 0.84 | 0.79 | 0.73 | 0.31 | 0.00 | 0.03 |
| Metabolism of cofactors and vitamins | 0.03 | 0.03 | 0.02 | 0.39 | 0.00 | 0.13 |
| NOD-like receptor signaling pathway | 0.1 | 0.08 | 0.07 | 0.31 | 0.00 | 0.31 |
| Nicotinate and nicotinamide metabolism | 0.85 | 0.87 | 0.89 | 0.70 | 0.00 | 0.48 |
| Nitrotoluene degradation | 0.07 | 0.07 | 0.08 | 0.59 | 0.00 | 0.06 |
| Nucleotide excision repair | 0.74 | 0.74 | 0.72 | 0.31 | 0.00 | 0.39 |
| Nucleotide metabolism | 0.27 | 0.26 | 0.24 | 0.59 | 0.01 | 0.18 |
| Other glycan degradation | 0.27 | 0.28 | 0.29 | 0.31 | 0.01 | 0.31 |
| ABC transporters | 0.74 | 0.72 | 0.7 | 0.24 | 0.01 | 0.48 |
| PPAR signaling pathway | 0.11 | 0.11 | 0.12 | 0.82 | 0.01 | 0.48 |
| Parkinson's disease | 0.01 | 0.02 | 0.02 | 0.03 | 0.01 | 0.59 |
| Pentose and glucuronate interconversions | 1.11 | 1.14 | 1.14 | 0.48 | 0.01 | 0.94 |
| Peptidases | 0.17 | 0.17 | 0.19 | 0.59 | 0.02 | 0.06 |
| Peroxisome | 0.41 | 0.41 | 0.43 | 0.82 | 0.02 | 0.06 |
| Phenylalanine tyrosine and tryptophan biosynthesis | 1.4 | 1.38 | 1.28 | 0.94 | 0.02 | 0.06 |
| Phenylpropanoid biosynthesis | 0.58 | 0.6 | 0.62 | 0.31 | 0.02 | 0.18 |
| Polycyclic aromatic hydrocarbon degradation | 0.3 | 0.31 | 0.32 | 0.82 | 0.02 | 0.18 |
| Porphyrin and chlorophyll metabolism | 0.08 | 0.08 | 0.09 | 0.39 | 0.02 | 0.31 |
| Propanoate metabolism | 0.08 | 0.08 | 0.08 | 0.59 | 0.02 | 0.31 |
| Protein digestion and absorption | 0.49 | 0.52 | 0.53 | 0.06 | 0.02 | 0.39 |
| Protein export | 0.05 | 0.06 | 0.06 | 0.48 | 0.02 | 0.39 |
| Protein folding and associated processing | 0.04 | 0.04 | 0.05 | 0.13 | 0.02 | 0.48 |
| Proximal tubule bicarbonate reclamation | 0.92 | 0.93 | 0.94 | 0.39 | 0.02 | 0.48 |
| Pyruvate metabolism | 0.05 | 0.05 | 0.05 | 0.82 | 0.02 | 0.48 |
| Replication recombination and repair proteins | 0.05 | 0.06 | 0.06 | 0.18 | 0.02 | 0.70 |
| Secretion system | 0.02 | 0.03 | 0.03 | 0.31 | 0.02 | 0.82 |
| Sphingolipid metabolism | 1.42 | 1.38 | 1.34 | 0.48 | 0.03 | 0.04 |
| Starch and sucrose metabolism | 0.18 | 0.18 | 0.2 | 0.82 | 0.03 | 0.04 |
| Stilbenoid diarylheptanoid and gingerol biosynthesis | 0.25 | 0.25 | 0.28 | 0.94 | 0.03 | 0.18 |
| Sulfur relay system | 0.2 | 0.2 | 0.21 | 0.82 | 0.03 | 0.24 |
| Taurine and hypotaurine metabolism | 0.61 | 0.62 | 0.63 | 0.39 | 0.03 | 0.31 |
| Terpenoid backbone biosynthesis | 0.11 | 0.11 | 0.12 | 0.48 | 0.03 | 0.31 |
| Two-component system | 0.08 | 0.08 | 0.09 | 0.70 | 0.03 | 0.31 |
| Type II diabetes mellitus | 0 | 0 | 0 | 0.82 | 0.03 | 0.31 |
| Zeatin biosynthesis | 0.11 | 0.11 | 0.11 | 0.13 | 0.03 | 0.39 |
| Adipocytokine signaling pathway | 0.06 | 0.05 | 0.05 | 0.18 | 0.03 | 0.39 |
| Butirosin and neomycin biosynthesis | 0.76 | 0.8 | 0.81 | 0.04 | 0.03 | 0.48 |
| Carbohydrate digestion and absorption | 0.15 | 0.16 | 0.16 | 0.24 | 0.03 | 0.59 |
| Cardiac muscle contraction | 0.06 | 0.06 | 0.06 | 0.18 | 0.03 | 0.82 |
| Cell cycle - Caulobacter | 0.54 | 0.56 | 0.56 | 0.18 | 0.03 | 0.94 |
| Alanine aspartate and glutamate metabolism | 0.65 | 0.66 | 0.66 | 0.39 | 0.03 | 0.94 |
| Cellular antigens | 0.09 | 0.09 | 0.09 | 0.59 | 0.04 | 0.09 |
| Chlorocyclohexane and chlorobenzene degradation | 0.11 | 0.11 | 0.12 | 0.94 | 0.04 | 0.13 |
| Cyanoamino acid metabolism | 0.95 | 0.95 | 0.97 | 0.94 | 0.04 | 0.31 |
| Cysteine and methionine metabolism | 1.88 | 1.92 | 1.92 | 0.13 | 0.04 | 0.70 |
| D-Arginine and D-ornithine metabolism | 2.77 | 2.7 | 2.66 | 0.59 | 0.04 | 0.70 |
| Amino acid metabolism | 0.86 | 0.91 | 0.92 | 0.04 | 0.04 | 0.82 |
| Drug metabolism - other enzymes | 1.55 | 1.49 | 1.42 | 0.59 | 0.04 | 0.82 |
| Electron transfer carriers | 0.48 | 0.49 | 0.49 | 0.59 | 0.04 | 1.00 |
